# COVID-19 Vaccine Uptake among Healthcare Workers: A Systematic Review and Meta-Analysis

**DOI:** 10.3390/vaccines10101637

**Published:** 2022-09-29

**Authors:** Petros Galanis, Irene Vraka, Aglaia Katsiroumpa, Olga Siskou, Olympia Konstantakopoulou, Theodoros Katsoulas, Theodoros Mariolis-Sapsakos, Daphne Kaitelidou

**Affiliations:** 1 Clinical Epidemiology Laboratory, Faculty of Nursing, National and Kapodistrian University of Athens, 11527 Athens, Greece; 2 Department of Radiology, P. & A. Kyriakou Children’s Hospital, 11527 Athens, Greece; 3 Department of Tourism Studies, University of Piraeus, 18534 Piraeus, Greece; 4 Center for Health Services Management and Evaluation, Faculty of Nursing, National and Kapodistrian University of Athens, 11527 Athens, Greece; 5 Faculty of Nursing, National and Kapodistrian University of Athens, 11527 Athens, Greece

**Keywords:** COVID-19, vaccination, healthcare workers, predictors

## Abstract

The vaccine-induced immunity of healthcare workers (HCWs) is crucial to controlling the COVID-19 pandemic. Therefore, we conducted a systematic review and meta-analysis to assess the COVID-19 vaccine uptake among HCWs worldwide and to identify predictors of vaccination. We searched Scopus, Web of Science, Medline, PubMed, ProQuest, CINAHL, and medRxiv up to 25 August 2022. We applied the Preferred Reporting Items for Systematic Reviews and Meta-Analysis guidelines. We found 25 studies including 491,624 vaccinated HCWs, while the full sample included 555,561 HCWs. The overall proportion of vaccinated HCWs was 77.3%. Vaccine uptake for studies that were conducted in North America (85.6%) was higher than the proportion for studies that were conducted in Asia (79.5%), Europe (72.8%), and Africa (65.6%). The overall prevalence of COVID-19 vaccine uptake was 83.6% and 77.4% for physicians and nurses, respectively. Older age, white race, physicians’ profession, seasonal influenza vaccine, direct COVID-19 patient care, and confidence in COVID-19 vaccine safety and effectiveness were positive predictors of vaccine uptake, while history of SARS-CoV-2 infection was a negative predictor. Deep understanding of the factors that influence HCWs’ decisions to receive a COVID-19 vaccine is critical to implementing tailored communication strategies for HCWs who are at risk for not getting vaccinated.

## 1. Introduction

The emergence and spread of SARS-CoV-2 has caused significant morbidity and mortality across the globe. As of August 2022, the number of total worldwide confirmed COVID-19 cases is estimated to be over 600 million, and over 6.5 million COVID-19-related deaths have been recorded [1]. Moreover, the COVID-19 pandemic continuous to threaten public health since breakthrough infections and new contagious variants of SARS-CoV-2 affect even fully vaccinated individuals [2,3]. 

The impact of the pandemic on healthcare workers (HCWs) has been unprecedented; between 80,000 and 180,000 HCWs died from COVID-19 between January 2020 and May 2021 according to WHO data [4]. HCWs are at increased risk of COVID-19 and hospitalization due to close and long contact with COVID-19 patients and occupational exposure to SARS-CoV-2 [5]. “Front-door” and patient-facing HCWs are at higher risk for COVID-19-related outcomes [6,7]. According to a meta-analysis with 119,883 HCWs, 51.7% of HCWs became infected with SARS-CoV-2 [8], while another meta-analysis with 230,398 HCWs found that 5% of COVID-19 cases in HCWs had severe complications, and 0.5% of HCWs died [9].

Globally, HCWs were among the first groups to be offered the COVID-19 vaccines since they are a high-risk group during the pandemic. Although safety and effectiveness of COVID-19 vaccines are proven both in randomized clinical trials [10,11] and real-world studies [12,13], COVID-19 vaccination hesitancy in HCWs worldwide continues to be a challenge [14,15,16]. For instance, the average prevalence of COVID-19 vaccination hesitancy worldwide in a total sample of 76,471 HCWs from 21 countries was 22.51%, ranging from 4.3 to 72% [14]. Sociodemographic factors (female gender, younger age, nurse profession, absence of history of influenza vaccination), concerns about vaccine safety and effectiveness, fear of adverse events, lack of information about vaccination, and mistrust of government and institutions are associated with COVID-19 vaccination hesitancy [14,17,18].

COVID-19 vaccination of healthcare workers is important both for protection of the individuals from SARS-CoV-2 and as an infection control practice to reduce nosocomial transmission of the virus since a significant proportion of COVID-19 inpatients acquire their infection in hospital [19,20]. A great number of studies have already investigated the intention of HCWs to accept a COVID-19 vaccine, finding low pooled acceptance rates: 63.5% in a meta-analysis with 50,940 HCWs worldwide [21] and 78% in a meta-analysis with 45,760 HCWs in China [22].

Several systematic reviews and meta-analyses investigated intention of HCWs to accept a COVID-19 vaccine [21,22,23,24,25]. Evidence shows that the main predictors for COVID-19 vaccine acceptance among HCWs are male gender, older age, and previous influenza vaccination. On the other hand, concerns about vaccine safety, efficacy, and effectiveness and limited information are the main barriers for vaccine acceptance. Moreover, several studies investigated COVID-19 vaccine uptake among HCWs. These studies were conducted mainly in the USA, Europe, and Africa. However, until now, no systematic review and meta-analysis have been published on this subject. Therefore, the aim of our study was to synthesize the available evidence about the COVID-19 vaccination uptake of HCWs. We conducted a systematic review and meta-analysis to assess the overall COVID-19 vaccine uptake among HCWs and to identify predictors of vaccination.

## 2. Materials and Methods

### 2.1. Data Sources and Strategy

We applied the Preferred Reporting Items for Systematic Reviews and Meta-Analysis (PRISMA) guidelines to perform this systematic review and meta-analysis [26]. We searched Scopus, Web of Science, Medline, PubMed, ProQuest, CINAHL, and medRxiv from inception to 25 August 2022. We used the following strategy in all fields: (((“healthcare worker *” OR “health care worker *” OR HCW OR HCWs OR “healthcare professional *” OR “health care professional *” OR HCPs OR “health care personnel” OR “healthcare personnel” OR “health personnel” OR HCP OR staff OR doctor * OR physician * OR clinician * OR nurs * OR “nursing staff” OR midwives OR midwife * OR paramedic * OR worker * OR professional * OR employee * OR personnel OR hospital * OR practitioner *) AND (vaccin *)) AND (uptake)) AND (COVID-19 OR COVID19 OR COVID OR SARS-CoV * OR “Severe Acute Respiratory Syndrome Coronavirus *” OR coronavirus *).

### 2.2. Selection and Eligibility Criteria

Two independent researchers performed the systematic literature search, and a third senior researcher resolved the differences. First, we removed duplicates and then we screened titles, abstracts, and full texts excluding articles which did not meet our inclusion criteria. Inclusion criteria for our review and meta-analysis were (a) quantitative studies reporting HCWs who received at least one dose of a COVID-19 vaccine, (b) studies including HCWs who have been providing healthcare during the COVID-19 pandemic, (c) individual studies with original data, and (d) studies published in English. Exclusion criteria were (a) studies reporting COVID-19 vaccine uptake on populations other than HCWs (e.g., the general population, students, and other occupations); (b) studies that did not separate vaccinated HCWs from HCWs intended to be vaccinated; and (c) reviews, qualitative studies, clinical cases, study protocols, conferential proceedings, commentaries, letters to the Editor, expert opinions, and editorials.

### 2.3. Data Extraction

Three researchers independently extracted the following data from the studies: first author and year of publication, country, data collection time, sample size, gender (percentage of females), age, study design, sampling method, recruitment method, response rate, type of publication (journal or pre-print service), and predictors of COVID-19 vaccine uptake among HCWs.

### 2.4. Risk of Bias (Quality) Assessment

We used the Joanna Briggs Institute critical appraisal tool to assess the risk of bias [27]. There are eight domains of bias and four possible answers: “Yes” when the criteria are clearly identifiable through the article; “No” when the criteria are not identifiable; “Unclear” when the criteria are not clearly identified in the article; and “Not applicable” when the criteria do not apply to the study. We used the “Yes” responses to rank the risk of bias as “low”, “moderate”, or “high”.

### 2.5. Statistical Analysis

We measured the overall COVID-19 vaccine uptake among HCWs in our meta-analysis. For each study, we divided the number of vaccinated HCWs by the total number of HCWs to calculate the proportion of HCWs that received at least one dose of a COVID-19 vaccine. In a similar way, we calculated the proportion of physicians and nurses that took a COVID-19 vaccine since the majority of studies reported detailed data (number of vaccinated physicians/nurses and total number of physicians/nurses) only for physicians and nurses. Then, we used the Freeman–Tukey double arcsine method to transform the above proportions and to calculate the overall COVID-19 vaccination proportion among HCWs, physicians, and nurses [28]. We calculated the 95% confidence intervals (CIs) for the proportions. 

Heterogeneity among studies was assessed using the I2 index (with an I2 > 75% representing high heterogeneity) and the Hedges Q statistic (with a *p*-value < 0.1 indicating statistically significant heterogeneity) [29]. Since the heterogeneity between results in our meta-analysis was very high, we applied random effects models [29]. In order to identify sources of heterogeneity, we considered performing subgroup analysis for continent of the study, study design, sampling method, recruitment method, type of publication, and quality of studies. However, we could not perform subgroup analysis for the sampling method and quality of studies due to the limited variability of these variables. Moreover, we used data collection time (giving the number 1 for studies that were conducted in January 2021, the number 2 for studies that were conducted in February 2021, etc.), sample size, gender (percentage of females), age (mean value), and response rate as independent variables in meta-regression models. We conducted a leave-one-out sensitivity analysis to assess the impact of each study on the overall COVID-19 vaccine uptake among HCWs.

We inspected funnel plots and assessed Egger’s test (with a *p*-value < 0.05 representing publication bias) to assess publication bias [30].

There was a high heterogeneity in the way that authors investigated the predictors of COVID-19 vaccine uptake among HCWs. For example, authors used different cut-off points for continuous variables such as age. Therefore, we did not conduct a meta-analysis, but we presented the impact of predictors by measuring the percentage of studies finding positive, negative, or no significant relationships (*p*-value < 0.05). We performed a quantitative analysis between predictors and COVID-19 vaccine uptake among HCWs. In that case, we presented measures of effect (e.g., odds ratios) and measures of precision (e.g., confidence intervals) that quantify the association between predictors and COVID-19 uptake in order to provide more information.

All analyses were conducted using OpenMeta [Analyst] (Brown University, Rhode Island, USA) [31].

## 3. Results

### 3.1. Identification and Selection of Studies

**Figure 1:**
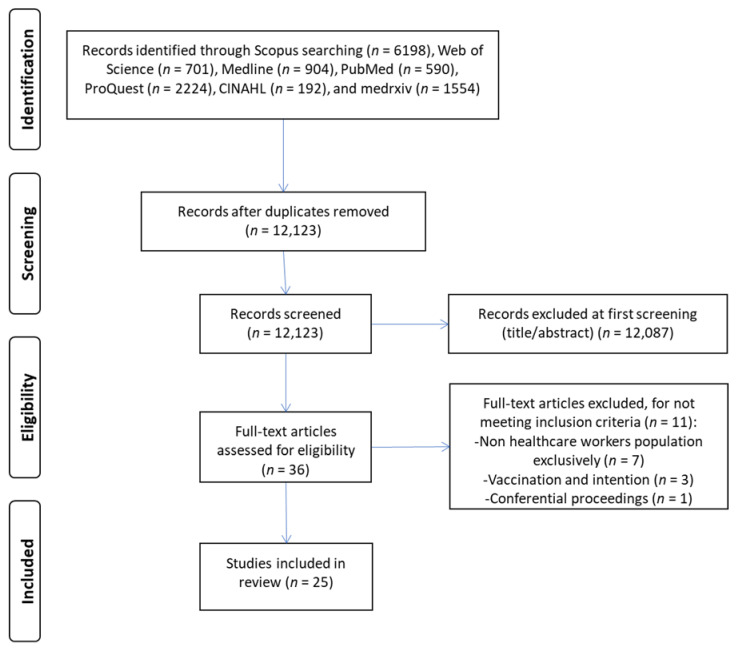
Flowchart of the literature search according to the PRISMA guidelines.

The initial search yielded 12,123 records after removal of duplicates. We assessed the full texts of 36 articles, and we excluded 11 applying our inclusion and exclusion criteria. Finally, we included 25 studies in our systematic review and meta-analysis [32,33,34,35,36,37,38,39,40,41,42,43,44,45,46,47,48,49,50,51,52,53,54,55,56]. The flow diagram according to PRISMA guidelines is shown in Figure 1.

### 3.2. Characteristics of the Studies

Main characteristics of the studies included in our review are presented in Table S1. We found 25 studies including 491,624 vaccinated HCWs, while the full sample included 555,561 HCWs, ranging from 172 to 356,053 [32,33,34,35,36,37,38,39,40,41,42,43,44,45,46,47,48,49,50,51,52,53,54,55,56]. The mean percentage of females was 67.3% (standard deviation = 19.9), and the mean age of the full sample was 39.2 years (standard deviation = 6.2). Studies included HCWs from 17 countries in five continents: eight (32%) from North America, six (24%) from Europe, six (24%) from Africa, four (16%) from Asia and one (4%) from South America. Authors collected data from January 2021 to February 2022 using online surveys (*n* = 12, 48%), paper surveys (*n* = 9, 36%), or administrative/registry data (*n* = 4, 16%). All studies were cross-sectional. Twenty-one studies (84%) were published in journals and four studies (16%) in pre-print services.

### 3.3. Risk of Bias (Quality) Assessment

Risk of bias was low in 24 studies and moderate in one study [42]. Detailed scores for each individual study are shown in Table S2.

### 3.4. COVID-19 Vaccine Uptake

#### 3.4.1. All Healthcare Workers

**Figure 2:**
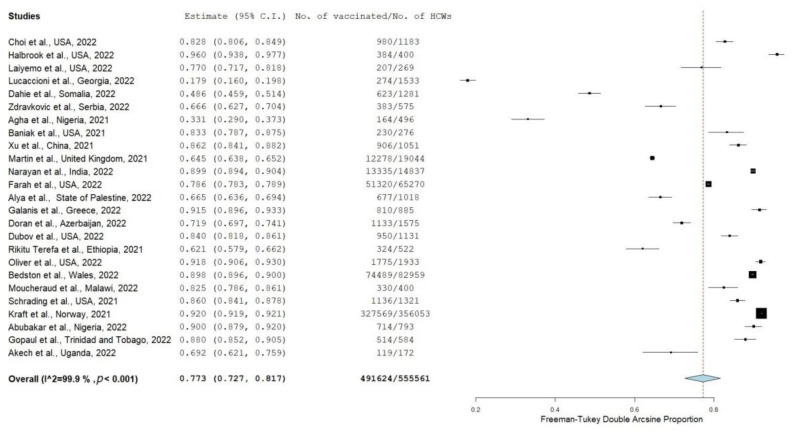
Forest plot of healthcare workers vaccinated against COVID-19 [32,33,34,35,36,37,38,39,40,41,42,43,44,45,46,47,48,49,50,51,52,53,54,55,56].

COVID-19 vaccine uptake among all HCWs was reported in 25 studies, including a total sample of 555,561 HCWs. Detailed results of COVID-19 vaccine uptake among healthcare workers are displayed in Table S3. The overall proportion of vaccinated HCWs was 77.3% (95% CI: 72.7–81.7%) (Figure 2). COVID-19 vaccination prevalence varied substantially from 17.9% [35] to 96.0% [33]. The heterogeneity was very high (I2 = 99.9%, *p*-value for the Hedges Q statistic < 0.001). Leave-one-out sensitivity analysis revealed that no single study showed a disproportionate impact on the uptake prevalence, which varied between 76.3% (95% CI: 71.5–80.9%) and 79.6% (95% CI: 75.3–83.5%) (Figure S1). Publication bias was not probable according to Egger’s test (>0.05) and the funnel plot (Figure S2).

**Figure 3:**
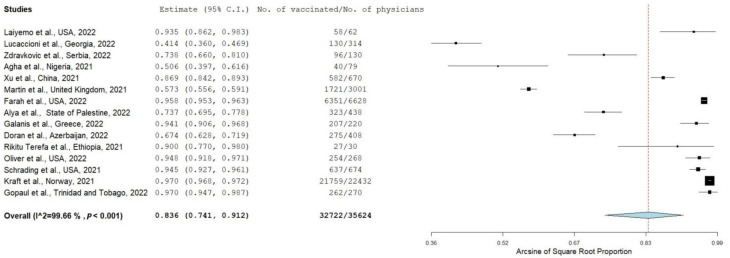
Forest plot of physicians vaccinated against COVID-19 [34,35,37,38,40,41,43,44,45,46,48,49,52,53,55].

Vaccine uptake for studies that were conducted in North America (85.6% (95% CI: 80.5–90.0%), I2 = 98.49) was higher than the proportion for studies that were conducted in Asia (79.5% (95% CI: 66.2–90.2%), I2 = 99.5), Europe (72.8% (95% CI: 62.2–82.2%), I2 = 99.9), and Africa (65.6% (95% CI: 45.6–83.0%), I2 = 99.3) (Figure 3). We did not find other substantial differences according to the other subgroup analyses. Meta-regression analysis did not find statistically significant differences regarding data collection time (*p* = 0.064), sample size (*p* = 0.391), percentage of females (*p* = 0.394), or mean age (*p* = 0.419).

#### 3.4.2. Physicians

COVID-19 vaccine uptake among physicians was reported in 15 studies, including a total sample of 35,624 physicians. Vaccination prevalence among physicians was 83.6% (95% CI: 74.1–91.2%), ranging from 41.4% [35] to 97.0% [53,55] (Figure 3). We found a very high heterogeneity between results (I2 = 99.7%, *p*-value for the Hedges Q statistic < 0.001). According to leave-one-out sensitivity analysis, no single study showed a disproportionate impact on the uptake prevalence, which varied between 82.2% (95% CI: 69.9–91.9%) and 85.9% (95% CI: 77.1–92.9%) (Figure S4). Publication bias was probable according to Egger’s test (<0.05) and the funnel plot (Figure S5).

Vaccine uptake for studies that were conducted in North America (95.5% (95% CI: 94.9–96.2%), I2 = 10.2) was higher than the proportion for studies that were conducted in Asia (76.6% (95% CI: 63.4–87.6%), I2 = 96.9), Europe (76.3% (95% CI: 45.0–96.8%), I2 = 99.9), and Africa (72.1% (95% CI: 28.8–98.8%), I2 = 94.5) (Figure S6). We did not find other substantial differences according to the other subgroup analyses. Meta-regression analysis did not find statistically significant differences regarding data collection time (*p* = 0.719), sample size (*p* = 0.636), percentage of females (*p* = 0.850), or mean age (*p* = 0.407).

#### 3.4.3. Nurses

**Figure 4:**
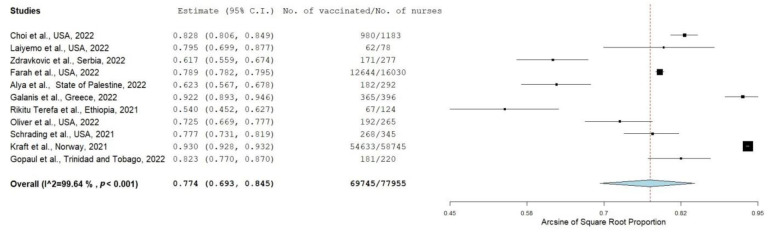
Forest plot of nurses vaccinated against COVID-19 [32,34,37,43,44,45,48,49,52,53,55].

COVID-19 vaccine uptake among nurses was reported in 11 studies, including a total sample of 77,955 nurses. COVID-19 vaccination prevalence among nurses was 77.4% (95% CI: 69.3–84.5%) (Figure 4). The overall proportion of vaccinated nurses varied substantially from 54.0% [48] to 93.0% [53]. The heterogeneity was very high (I2 = 99.6%, *p*-value for the Hedges Q statistic < 0.001). Leave-one-out sensitivity analysis revealed that no single study showed a disproportionate impact on the uptake prevalence, which varied between 75.5% (95% CI: 66.8–83.3%) and 79.3% (95% CI: 71.2–86.4%) (Figure S7). Egger’s test (<0.05) and the funnel plot revealed a probable publication bias (Figure S8).

Vaccine uptake for studies that were conducted in Europe (84.5% (95% CI: 67.1–96.1%), I2 = 98.9) was higher than the proportion for studies that were conducted in North America (78.8% (95% CI: 75.8–81.6%), I2 = 77.8) (Figure S9). Further subgroup analyses did not find any other substantial differences. Moreover, meta-regression analysis did not find statistically significant differences regarding data collection time (*p* = 0.569), sample size (*p* = 0.703), percentage of females (*p* = 0.103), or mean age (*p* = 0.069).

### 3.5. Predictors of COVID-19 Vaccine Uptake among Healthcare Workers

Twenty-one studies investigated predictors of COVID-19 vaccine uptake among HCWs [32,34,35,36,37,38,39,40,41,43,44,45,46,47,48,49,50,51,54,55,56]. Detailed results of the predictors are presented in Table 1 and Table 2. Authors investigated sociodemographic characteristics of the HCWs, COVID-19-related variables, and COVID-19 vaccine-related variables as possible predictors of vaccine uptake among HCWs. In particular, older age, white race, physicians’ profession, and seasonal influenza vaccine were positive predictors in 12/18, 4/7, 8/16, and 4/4 studies, respectively. Moreover, direct COVID-19 patient care (4/7 studies) and confidence in COVID-19 vaccine safety (6/6 studies) were associated with increased vaccine uptake, while history of SARS-CoV-2 infection (6/9 studies) was associated with decreased vaccine uptake. Gender, marital status, educational level, income, work experience, chronic condition, severity perception of COVID-19, self-rated health status, and knowledge and information about COVID-19 vaccines were not predictors of vaccine uptake (10/17, 3/4, 3/6, 2/3, 3/3, 7/7, 2/3, 2/3, 2/2, and 2/2 studies, respectively, found no relationship). Moreover, we quantified the relationship between predictors and COVID-19 vaccine uptake among HCWs presenting statistically significant odds ratios (Table S4). There was a wide range in values of odds ratios among studies. For instance, HCWs that trust COVID-19 vaccines were 1.43 to 7.48 times more likely to take a vaccine. The odds of physicians versus the odds of other HCWs to receive a vaccine ranged from 1.80 to 11.11.

**Table 1:** Predictors of COVID-19 vaccine uptake among healthcare workers.

Reference	Older Age	Males	Married	Higher Educational Level	White Race	Higher Income	Physicians	Work Experience	Chronic Condition
Choi et al. [32]	-	-	-	-	↓	-	-	-	-
Laiyemo et al. [34]	↑	NS	NS	↑	NS	-	NS	-	-
Lucaccioni et al. [35]	↑	NS	-	-	-	-	↑	-	NS
Dahie et al. [36]	↑	↑	-	↑	-	-	↑	-	NS
Zdravkovic et al. [37]	↑	↓	-	-	-	-	↑	NS	-
Agha et al. [38]	-	-	-	↑	-	-	↑	-	-
Baniak et al. [39]	NS	NS	NS	-	NS	-	-	NS	-
Xu et al. [40]	NS	NS	-	NS	-	-	NS	-	-
Martin et al. [41]	↑	↑	-	-	↑	-	↓	-	-
Farah et al. [43]	↑	↑	-	-	↑	-	↑	-	-
Alya et al. [44]	-	↑	↓	-	-	↑	-	-	-
Galanis et al. [45]	NS	↓	NS	NS	-	NS	NS	NS	NS
Doran et al. [46]	↑	NS	-	-	-	-	NS	-	NS
Dubov et al. [47]	↓	-	-	-	-	-	-	-	-
Rikitu Terefa et al. [48]	↑	↑	-	NS	-	NS	NS	-	NS
Oliver et al. [49]	NS	NS	-	-	↑	-	↑	-	-
Bedston et al. [50]	↑	NS	-	-	↑	-	↑	-	-
Moucheraud et al. [51]	NS	NS	-	-	-	-	NS	-	NS
Abubakar et al. [54]	↑	NS	-	-	-	-	-	-	-
Gopaul et al. [55]	↑	NS	-	-	-	-	↑	-	-
Akech et al. [56]	↑	-	-	-	-	-	NS	-	NS
Positive association ^a^	12/18	5/17	0/4	3/6	4/7	1/3	8/16	0/3	0/7
Negative association ^b^	1/18	2/17	1/4	0/6	1/7	0/3	1/16	0/3	0/7
No association ^c^	5/18	10/17	3/4	3/6	2/7	2/3	7/16	3/3	7/7

^a^ number of studies with a positive significant association (*p*-value < 0.05) between the predictor and COVID-19 vaccine uptake/total number of studies examining the predictor. ^b^ number of studies with a negative significant association (*p*-value < 0.05) between the predictor and COVID-19 vaccine uptake/total number of studies examining the predictor. ^c^ number of studies without a significant association (*p*-value ≥ 0.05) between the predictor and COVID-19 vaccine uptake/total number of studies examining the predictor. NS: non-significant. ↑ more likely to accept. ↓ less likely to accept. - not investigated.

**Table 2:** Predictors of COVID-19 vaccine uptake among healthcare workers.

Reference	History of SARS-CoV-2 Infection	Higher Severity Perception of COVID-19	Direct COVID-19 Patient Care	Self-Rated Health Status	Influenza Vaccine	Confidence in COVID-19 Vaccine Effectiveness	Confidence in COVID-19 Vaccine Safety	Knowledge of COVID-19 Vaccines	Information about COVID-19 Vaccines
Lucaccioni et al. [35]	↓	-	NS	NS	↑	↑	-	-	NS
Dahie et al. [36]	-	-	↑	-	-	-	-	-	-
Zdravkovic et al. [37]	NS	-	-	-	-	-	-	-	-
Baniak et al. [39]	-	-	-	-	-	NS	↑	NS	NS
Xu et al. [40]	-	-	-	-	-	-	↑	-	-
Martin et al. [41]	↓	-	-	-	-	-	-	-	-
Farah et al. [43]	↓	-	↑	-	-	-	-	-	-
Alya et al. [44]	↓	-	-	-	-	-	↑	-	-
Galanis et al. [45]	NS	NS	NS	-	↑	↑	↑	-	-
Doran et al. [46]	↓	NS	↑	↑	↑	NS	↑	NS	-
Dubov et al. [47]	↓	-	-	-	-	-	-	-	-
Rikitu Terefa et al. [48]	-	-	↑	NS	-	-	-	-	-
Oliver et al. [49]	-	-	-	-	↑	-	↑	-	-
Abubakar et al. [54]	NS	-	-	-	-	-	-	-	-
Gopaul et al. [55]	-	-	-	-	-	NS	-	-	-
Akech et al. [56]	-	↑	NS	-	-	-	-	-	-
Positive association ^a^	0/9	1/3	4/7	1/3	4/4	2/5	6/6	0/2	0/2
Negative association ^b^	6/9	0/3	0/7	0/3	0/4	0/5	0/6	0/2	0/2
No association ^c^	3/9	2/3	3/7	2/3	0/4	3/5	0/6	2/2	2/2

^a^ number of studies with a positive significant association (*p*-value < 0.05) between the predictor and COVID-19 vaccine uptake/total number of studies examining the predictor. ^b^ number of studies with a negative significant association (*p*-value < 0.05) between the predictor and COVID-19 vaccine uptake/total number of studies examining the predictor. ^c^ number of studies without a significant association (*p*-value ≥ 0.05) between the predictor and COVID-19 vaccine uptake/total number of studies examining the predictor. NS: non-significant. ↑ more likely to accept. ↓ less likely to accept. - not investigated.

## 4. Discussion

This meta-analysis identified, for the first time, the prevalence of COVID-19 vaccine uptake among HCWs based on data from 555,561 HCWs worldwide. Moreover, we found several predictors of HCWs’ decisions to receive a COVID-19 vaccine. Specifically, we found that older HCWs, white HCWs, physicians, those who had received the seasonal influenza vaccine, HCWs who provide care to COVID-19 patients, and those who trust COVID-19 vaccines are more likely to have been vaccinated. On the other hand, prior SARS-CoV-2 was associated with a decrease in vaccine uptake. 

### 4.1. COVID-19 Vaccine Uptake

The overall prevalence of COVID-19 vaccine uptake among HCWs in our meta-analysis, 77.3%, is higher than that detected in the general population, 67.6% [57]. This finding is confirmed by the literature since HCWs are more likely than the general population to accept a COVID-19 vaccine [58,59]. Lack of scientific knowledge and low understanding of COVID-19 vaccines among non-healthcare workers could explain the higher acceptance rate of healthcare workers. 

Although our study showed a high COVID-19 vaccine uptake rate among HCWs, not all of them received a COVID-19 vaccine. In particular, an important percentage (22.7%) of the HCWs in our study refused vaccination despite availability and accessibility showing that HCWs are not immune to vaccine hesitancy. Indeed, the WHO defines vaccine hesitancy as 1 of the 10 most important threats to global health in 2019 [60]. It is necessary to increase the uptake of COVID-19 vaccination by HCWs since they provide care to COVID-19 patients, and high infection rate among HCWs could result in a significant reduction in this essential workforce. Moreover, vaccinated and well-informed HCWs are an important source of COVID-19 vaccine information and are more likely to recommend COVID-19 vaccination to families, colleagues, and their patients [61]. HCWs should inform individuals and especially patients about the safety and effectiveness of COVID-19 vaccination since their vaccine hesitancy may exacerbate patient hesitancy [62]. HCWs are trusted professionals and could serve as a role model of healthy behavior of the general population, improving vaccine coverage [63]. 

Our findings demonstrate the highest prevalence of COVID-19 vaccine uptake among HCWs in North America (85.6%) and the lowest prevalence among those in Africa (65.6%). A similar trend is observed in the vaccination of the general population worldwide since the percentage of population to have received at least one dose of a COVID-19 vaccine is 80% in North America and Asia, 69% in Europe, and 27% in Africa [57]. The majority of the countries in Africa missed the WHO targets: 40% of a country’s population should have been vaccinated by end December 2021, and 70% by mid-2022 [64]. Inconsistent supply of COVID-19 vaccines to Africa, low country-level preparedness against a pandemic, vaccine hesitancy, and distrust are the main reasons for the low vaccination uptake in Africa [64]. Africa’s low vaccination rate is a threat to global effectiveness of COVID-19 vaccines since new variants could emerge from populations with low vaccine coverage. Therefore, positive attitudes of HCWs in Africa towards COVID-19 vaccination is paramount since they are a professional trusted source of information, and their opinion could improve vaccination coverage [65].

### 4.2. Predictors of COVID-19 Vaccine Uptake

We found variation in vaccine uptake across races since white HCWs are more likely to receive a COVID-19 vaccine. Several studies in the general population confirm that COVID-19 vaccination uptake is higher among white individuals than ethnic minority groups and especially black individuals [66,67,68]. Reduced vaccine uptake in ethnic minority groups is not unique to COVID-19 since this phenomenon was also observed for the H1N1 flu pandemic in 2009 [69,70]. Ethnicity is interrelated with other barriers to COVID-19 vaccine uptake, such as lower educational level and socioeconomic status, religion, belief in conspiracy theories, and lack of trust in the government, healthcare systems, and employers [71,72,73]. These findings give significant cause for concern, since SARS-CoV-2 infection rate and COVID-19-related adverse outcomes are higher in these groups [6,74,75,76]. Thus, there is an urgent to implement targeted interventions in order to overcome barriers to COVID-19 vaccination in ethnic minority groups [72,73,77].

Our review identified that physicians were more likely to be vaccinated than other staff groups. Moreover, the overall prevalence of COVID-19 vaccine uptake among physicians in our meta-analysis, 83.6%, is higher than that detected for nurses, 77.4%. Concerns about the safety, effectiveness, and side effects of COVID-19 vaccines and lack of perceived need for vaccination are the main reasons for vaccine hesitancy among nurses [78,79]. Nurses’ unwillingness to accept a COVID-19 vaccine is concerning since they are among the most trusted HCWs, and their attitudes towards vaccination can influence patient vaccination decisions [80,81]. Moreover, nurses are the frontline HCWs that provide direct care to COVID-19 patients, and thus nurses’ occupational risk of exposure to SARS-CoV-2 is very high [82,83,84,85]. The situation is worse for the nurses in ethnic minority groups since they have already been disproportionately affected by the pandemic. For instance, Filipino nurses comprise 25% of COVID-19-related deaths among nurses in the USA, although they make up 4% of the nursing workforce in the country [86].

Confidence in COVID-19 vaccine safety and effectiveness was found to be a positive predictor of COVID-19 vaccine uptake in our systematic review. Negative attitudes towards COVID-19 vaccination, concerns for side effects and future effects, and lack of confidence in safety and effectiveness are more common among unvaccinated HCWs [44,87,88]. The novelty and the rapid production of COVID-19 vaccines worldwide could explain mistrust of HCWs [89]. It is reasonable that some HCWs may have delayed vaccination until new data emerged. Therefore, additional and updated information about safety and effectiveness of vaccines may increase vaccine acceptance among HCWs. Detailed data on safety and surveillance regarding the COVID-19 vaccines could help HCWs to overcome their fear and hesitancy.

We found that seasonal influenza vaccination is a positive predictor of COVID-19 vaccine uptake. Flu vaccination denotes a positive attitude of HCWs towards vaccines, and promotion of annual influenza vaccination may have a positive effect in future COVID-19 vaccine acceptance. A systematic review has already shown that influenza vaccination positively affects HWCs’ intention to receive a COVID-19 vaccine [21]. However, since the flu vaccination rate among HCWs is low [90,91], investment and promotion of seasonal influenza vaccination is crucial.

According to our systematic review, direct care for COVID-19 patients was associated with vaccine uptake among HCWs. The literature supports this finding since a scoping review of 35 studies with 16,158 HCWs found that HCWs that provide care to COVID-19 patients are more likely to accept COVID-19 vaccines [14]. Direct patient care may be associated with higher severity perception of COVID-19, which drives nurses to be vaccinated more often [56].

It is not surprising that older age was a positive predictor of COVID-19 vaccine uptake among HCWs in our study. Early studies in the general population of the USA confirm this finding since vaccination coverage is lower and increased more slowly over time among younger adults [67,92]. Older age is a well-known significant predictor of COVID-19 mortality [93,94]. Thus, older adults may feel more vulnerable to COVID-19, which probably encourages them to take a COVID-19 vaccine considering health benefits. On the other hand, younger adults may perceive a lower personal risk of COVID-19-related outcomes.

Finally, we found that a prior SARS-CoV-2 infection is associated with a decrease in COVID-19 vaccine uptake. This finding can be explained by the fact that HCWs with a confirmed SARS-CoV-2 infection may believe that they have acquired sufficient immunological protection [41]. However, waning humoral immunity to SARS-CoV-2 in those who have had confirmed COVID-19 [95] and new variants of SARS-CoV-2 [2] threaten people’s immunization and increase risk of infection over time.

### 4.3. Limitations

Our systematic review and meta-analysis have several limitations. First, heterogeneity in the results of the meta-analysis was very high. We performed subgroup analysis and meta-regression analysis for several variables to overcome this bias, but it is impossible to address all possible sources of heterogeneity. Moreover, data for certain variables were limited. For example, some authors have not presented the mean age of the samples, but they have used different cut-off points to create age groups. Therefore, age data for these studies cannot be included in the meta-regression analysis. Second, authors mainly investigated the sociodemographic characteristics of HCWs as possible predictors of vaccine uptake. Thus, further studies should be conducted in order to investigate in-depth the predictors of vaccine uptake, e.g., psychological factors and social media factors. Third, studies were conducted mainly in the USA, Europe, and Africa, and thus we cannot generalize the results. However, we conducted a subgroup analysis in order to define the vaccine uptake in the different continents. Fourth, studies were conducted from January 2021 to February 2022. Since knowledge of COVID-19 vaccines and new variants of SARS-CoV-2 is increasing significantly on an ongoing basis, further studies should be conducted in order to capture the attitudes of HCWs in the future. Fifth, all studies in our review were cross-sectional, and thus causal relationships between predictors and vaccine uptake cannot arise. Moreover, cohort studies reporting changes in HCWs’ attitudes over time should add valuable evidence. Finally, only 2 out of 25 studies in our review used nationally representative samples, and thus a selection bias is probable in our review.

## 5. Conclusions

This systematic review and meta-analysis of HCWs’ decisions to take a COVID-19 vaccine found that 77.3% out of 555,561 HCWs worldwide have received at least one dose of a COVID-19 vaccine. The overall prevalence of COVID-19 vaccine uptake was 83.6% and 77.4% for physicians and nurses, respectively. Being older, white, or a physician; receiving the seasonal influenza vaccine; providing care to COVID-19 patients; and trusting COVID-19 vaccines were enabling factors for the COVID-19 vaccine uptake. Deep understanding of the factors that influence HCWs’ decisions to receive a COVID-19 vaccine is critical to implementing tailored communication strategies for HCWs who are at risk for not getting vaccinated, e.g., HCWs who belong to ethnic minority groups.

Since 22.7% of the HCWs in our full sample refused to receive a COVID-19 vaccine, education and policy-based interventions are needed in order to improve the vaccination coverage among HCWs. In particular, transparency of data on the safety and efficacy of vaccines is seen as key to improving vaccine uptake. HCWs should play a pivotal role in the acceptance of COVID-19 vaccination by the general population. Low compliance for COVID-19 vaccination among HCWs is an enormous risk for healthcare systems and public health. HCWs are frontline workers against COVID-19 experiencing high levels of burnout and mental health issues during the pandemic. Thus, vaccination of HCWs is an ethical duty of healthcare systems. Since COVID-19 vaccine policymaking is a complex and unique issue, multi-component interventions (e.g., tailored messages, incentives, educational material, on-site vaccination, minimization of inequities, financial and social support) seem to be an effective strategy to improve vaccination coverage among HCWs.

## Data Availability

The data presented in this study are available on request from the corresponding author.

## Supplementary Materials

The following supporting information can be downloaded at: https://www.mdpi.com/article/10.3390/vaccines10101637/s1, Figure S1: Leave-one-out sensitivity analysis for healthcare workers vaccinated against COVID-19; Figure S2: Funnel plot of healthcare workers vaccinated against COVID-19; Figure S3: Forest plot of healthcare workers vaccinated against COVID-19 according to the continent that studies were conducted; Figure S4: Leave-one-out sensitivity analysis for physicians vaccinated against COVID-19; Figure S5: Funnel plot of physicians vaccinated against COVID-19; Figure S6: Forest plot of physicians vaccinated against COVID-19 according to the continent that studies were conducted; Figure S7: Leave-one-out sensitivity analysis for nurses vaccinated against COVID-19; Figure S8: Funnel plot of nurses vaccinated against COVID-19; Figure S9: Forest plot of nurses vaccinated against COVID-19 according to the continent that studies were conducted; Table S1: Overview of the studies included in systematic review. Table S2: Quality of studies included in this systematic review; Table S3: COVID-19 vaccine uptake among healthcare workers. Table S4: Measures of effect and precision between predictors and COVID-19 vaccine uptake among healthcare workers. Table S5: Measures of effect and precision between predictors and COVID-19 vaccine uptake among healthcare workers [32,33,34,35,36,37,38,39,40,41,42,43,44,45,46,47,48,49,50,51,52,53,54,55,56].
