# Supplementary material for: COVID-19 Vaccine Uptake among Healthcare Workers: A Systematic Review and Meta-Analysis
Source: Vaccines (Basel). 2022 Sep 29;10(10):1637. doi: 10.3390/vaccines10101637 (PMC9610263; doi:10.3390/vaccines10101637)
Supplement: Supplementary file 1 [file vaccines-10-01637-s001.zip › Supplementary Table S1.pdf]

Supplementary Table S1. Overview of the studies included in systematic review.

| Reference | Country | Data collection time | Sample size (n) | Females (%) | Age, mean (standard deviation) | Study design | Sampling method | Recruitment method | Response rate (%) | Published in |
|---|---|---|---|---|---|---|---|---|---|---|
| (Choi et al., 2022) | USA | March to April 2021 | 1183 | 89.4 | 18-40 years, 32.7%; 41-60 years, 58.4%; >60 years, 8.8% | Cross-sectional | Convenience | Online survey | NA | Journal |
| (Halbrook et al., 2022) | USA | January to March 2021 | 400 | 70.2 | 18-39 years, 56.4%; 40-59 years, 36.9%; >59 years, 6.7% | Cross-sectional | Convenience | Paper survey | NR | Journal |
| (Laiyemo et al., 2022) | USA | January to March 2021 | 277 | 68.1 | 42.2 (13.8) | Cross-sectional | Convenience | Paper survey | 92.3 | Journal |
| (Lucaccioni et al., 2022) | Georgia | March to July 2021 | 1533 | 84.1 | 41 (29-53)[a] | Cross-sectional | Convenience | Paper survey | NR | Journal |
| (Dahie et al., 2022) | Somalia | December 2021 to February 2022 | 1281 | 49.2 | 27.7 (7.1) | Cross-sectional | Convenience | Online survey | NA | Journal |
| (Zdravkovic et al., 2022) | Serbia | January to September 2021 | 575 | 80.3 | 41.6 (11.3) | Cross-sectional | Convenience | Paper survey | 62.8 | Journal |
| (Agha et al., 2021) | Nigeria | July 2021 | 496 | 48.8 | 18-29 years, 30.4%; 30-39 years, 51.2%; >39 years, 18.3% | Cross-sectional | Convenience | Online survey | NA | Journal |
| (Baniak et al., 2021) | USA | February 2021 | 276 | 83.7 | 48.3 (10.2) | Cross-sectional | Convenience | Online survey | 21.2 | Journal |
| (Xu et al., 2021) | China | April 2021 | 1051 | 89.4 | 18-40 years, 55.4%; 41-50 years, 26.5%; >50 years, 18.1% | Cross-sectional | Convenience | Online survey | 96.7 | Journal |
| (Martin et al., 2021) | United Kingdom | February 2021 | 19,044 | 75.6 | 18-40 years, 47.8%; 41-60 years, 44.1%; >60 years, 8.1% | Cross-sectional | Convenience | Paper survey | NR | Journal |
| (Narayan et al., 2022) | India | NR | 14,837 | 51.1 | 38.3 (11.7) | Cross-sectional | Convenience | Administrative/registry data | NA | Journal |
| (Farah et al., 2022) | USA | April 2021 | 65,270 | 71.0 | 40.0 (interquartile range, 16-100) | Cross-sectional | Convenience | Administrative/registry data | NA | Journal |
| (Alya et al., 2022) | State of Palestine | April to May 2021 | 1018 | 55.0 | <30 years, 38.8%; 30-50 years, 52.9%; >50 years, 8.3% | Cross-sectional | Convenience | Online survey | NA | Journal |
| (Galanis et al., 2022) | Greece | August 2021 | 885 | 80.7 | 40.9 (9.9) | Cross-sectional | Convenience | Online survey | NA | Journal |
| (Doran et al., 2022) | Azerbaijan | May to July 2021 | 1575 | 93.0 | 47.0 (NR) | Cross-sectional | Convenience | Paper survey | NR | Journal |

| | | | | | | | | | | |
|---|---|---|---|---|---|---|---|---|---|---|
| (Dubov et al., 2022) | USA | February to April 2021 | 1131 | 79.5 | 25-40 years, 48%; >40 years, 52% | Cross-sectional | Convenience | Online survey | 20.9 | Journal |
| (Rikitu Terefa et al., 2021) | Ethiopia | June 2021 | 522 | 8.8 | 30.9 (4.8) | Cross-sectional | Convenience | Online survey | NA | Journal |
| (Oliver et al., 2022) | USA | February 2021 | 1933 | 70.5 | <40 years, 45.1%; 40-59 years, 38.8%; >59 years, 12.9% | Cross-sectional | Convenience | Online survey | NA | Journal |
| (Bedston et al., 2022) | Wales | June 2021 | 82,959 | 77.6 | <40 years, 39.2%; 40-59 years, 50.3%; >59 years, 10.5% | Cross-sectional | National survey | Administrative/registry data | NA | Journal |
| (Moucheraud et al., 2022) | Malawi | March to May 2021 | 400 | 44.8 | 32 (interquartile range, 28-38) | Cross-sectional | Convenience | Paper survey | NR | Journal |
| (Schrading et al., 2021) | USA | January 2021 | 1321 | 62.4 | <40 years, 58.7%; 40-49 years, 22.1%; >49 years, 19.2% | Cross-sectional | Convenience | Paper survey | 90.7 | Journal |
| (Kraft et al., 2021) | Norway | August 2021 | 356,053 | 81.0 | 41 (13) | Cross-sectional | National survey | Administrative/registry data | NA | Pre-print |
| (Abubakar et al., 2022) | Nigeria | May 2021 | 793 | 34.6 | 18-39 years, 65.8%; 40-59 years, 33.1%; >59 years, 1.1% | Cross-sectional | Convenience | Online survey | NA | Pre-print |
| (Gopaul et al., 2022) | Trinidad and Tobago | August to October 2021 | 584 | 79.5 | 18-34 years, 44.9%; 35-54 years, 51.4%; >54 years, 3.8% | Cross-sectional | Convenience | Online survey | 43.2 | Pre-print |
| (Akech et al., 2022) | Uganda | September to October 2021 | 172 | 55.0 | 18-39 years, 76.0%; >39 years, 24.0% | Cross-sectional | Convenience | Paper survey | 45.0 | Pre-print |

References

Abubakar, A. T., Suleiman, K., Ahmad, S. I., Suleiman, S. Y., Ibrahim, U. B., Suleiman, B. A., Haladu, S. A., Al-Mustapha, A. I., & Abubakar, M. I. (2022). *Acceptance of COVID-19 vaccine among healthcare workers in Katsina state, Northwest Nigeria* [Preprint]. Public and Global Health. https://doi.org/10.1101/2022.03.20.22272677

Agha, S., Chine, A., Lalika, M., Pandey, S., Seth, A., Wiyeh, A., Seng, A., Rao, N., & Badshah, A. (2021). Drivers of COVID-19 Vaccine Uptake amongst Healthcare Workers (HCWs) in Nigeria. *Vaccines*, *9*(10), 1162. https://doi.org/10.3390/vaccines9101162

Akech, G. M., Kanyike, A. M., Nassozi, A. G., Aguti, B., Nakawuki, A. W., Kimbugwe, D., Kiggundu, J., Maiteki, R., Mukyala, D., Bongomin, F., Obakiro, S. B., Rebecca, N., & Iramiot, J. S. (2022). *COVID-19 Vaccination Uptake and Self-Reported Side Effects among Healthcare Workers in Mbale City Eastern Uganda* [Preprint]. Infectious Diseases (except HIV/AIDS). https://doi.org/10.1101/2022.07.11.22277490

Alya, W. A., Maraqa, B., Nazzal, Z., Odeh, M., Makhalfa, R., Nassif, A., & Aabed, M. (2022). COVID-19 vaccine uptake and its associated factors among Palestinian healthcare workers: Expectations beaten by reality. *Vaccine*, *40*(26), 3713–3719. https://doi.org/10.1016/j.vaccine.2022.05.026

Baniak, L. M., Luyster, F. S., Raible, C. A., McCray, E. E., & Strollo, P. J. (2021). COVID-19 Vaccine Hesitancy and Uptake among Nursing Staff during an Active Vaccine Rollout. *Vaccines*, *9*(8), 858. https://doi.org/10.3390/vaccines9080858

Bedston, S., Akbari, A., Jarvis, C. I., Lowthian, E., Torabi, F., North, L., Lyons, J., Perry, M., Griffiths, L. J., Owen, R. K., Beggs, J., Chuter, A., Bradley, D. T., de Lusignan, S., Fry, R., Richard Hobbs, F. D., Hollinghurst, J., Katikireddi, S. V., Murphy, S., … Lyons, R. A. (2022). COVID-19 vaccine uptake, effectiveness, and waning in 82,959 health care workers: A national prospective cohort study in Wales. *Vaccine*, *40*(8), 1180–1189. https://doi.org/10.1016/j.vaccine.2021.11.061

Choi, K., Rondinelli, J., Cuenca, E., Lewin, B., Chang, J., Luo, Y. X., Bronstein, D., & Bruxvoort, K. (2022). Race/Ethnicity Differences in COVID-19 Vaccine Uptake Among Nurses. *Journal of Transcultural Nursing*, *33*(2), 134–140. https://doi.org/10.1177/10436596211065395

Dahie, H. A., Mohamoud, J. H., Adam, M. H., Garba, B., Dirie, N. I., Sh. Nur, M. A., & Mohamed, F. Y. (2022). COVID-19 Vaccine Coverage and Potential Drivers of Vaccine Uptake among Healthcare Workers in SOMALIA: A Cross-Sectional Study. *Vaccines*, *10*(7), 1116. https://doi.org/10.3390/vaccines10071116

Doran, J., Seyidov, N., Mehdiyev, S., Gon, G., Kissling, E., Herdman, T., Suleymanova, J., Rehse, A. P. C., Pebody, R., Katz, M. A., & Hagverdiyev, G. (2022). Factors associated with early uptake of COVID-19 vaccination among healthcare workers in Azerbaijan, 2021. *Influenza and Other Respiratory Viruses*, *16*(4), 626–631. https://doi.org/10.1111/irv.12978

Dubov, A., Distelberg, B. J., Abdul-Mutakabbir, J. C., Peteet, B., Roberts, L., Montgomery, S. B., Rockwood, N., Patel, P., Shoptaw, S., & Chrissian, A. A. (2022). Racial/Ethnic Variances in COVID-19 Inoculation among Southern California Healthcare Workers. *Vaccines*, *10*(8), 1331. https://doi.org/10.3390/vaccines10081331

Farah, W., Breeher, L., Shah, V., Hainy, C., Tommaso, C. P., & Swift, M. D. (2022). Disparities in COVID-19 vaccine uptake among health care workers. *Vaccine*, *40*(19), 2749–2754. https://doi.org/10.1016/j.vaccine.2022.03.045

Galanis, P., Moisoglou, I., Vraka, I., Siskou, O., Konstantakopoulou, O., Katsiroumpa, A., & Kaitelidou, D. (2022). Predictors of COVID-19 Vaccine Uptake in Healthcare Workers: A Cross-Sectional Study in Greece. *Journal of Occupational & Environmental Medicine*, *64*(4), e191–e196. https://doi.org/10.1097/JOM.0000000000002463

Gopaul, C. D., Ventour, D., & Thomas, D. (2022). *COVID-19 Vaccine Acceptance and Uptake Among Healthcare Workers in Trinidad & Tobago* [Preprint]. Public and Global Health. https://doi.org/10.1101/2022.05.09.22274854

Halbrook, M., Gadoth, A., Martin-Blais, R., Gray, A. N., Kashani, S., Kazan, C., Kane, B., Tobin, N. H., Ferbas, K. G., Aldrovandi, G. M., & Rimoin, A. W. (2022). Longitudinal Assessment of Coronavirus Disease 2019 Vaccine Acceptance and Uptake Among Frontline Medical Workers in Los Angeles, California. *Clinical Infectious Diseases*, *74*(7), 1166–1173. https://doi.org/10.1093/cid/ciab614

Kraft, K. B., Elgersma, I., Lyngstad, T. M., Elstrøm, P., & Telle, K. (2021). *COVID-19 vaccination rates among health care workers by immigrant background. A nation-wide registry study from Norway* [Preprint]. Public and Global Health. https://doi.org/10.1101/2021.09.17.21263619

Laiyemo, A. O., Asemota, J., Deonarine, A., Aduli, F., & McDonald-Pinkett, S. (2022). Minority Healthcare Workers' Perception of Safety and COVID-19 Vaccination Uptake. *Journal of General Internal Medicine*, *37*(4), 1006–1007. https://doi.org/10.1007/s11606-021-07299-y

Lucaccioni, H., Chakhunashvili, G., McKnight, C. J., Zardiashvili, T., Jorgensen, P., Pebody, R., Kissling, E., Katz, M. A., & Sanodze, L. (2022). Sociodemographic and Occupational Factors Associated with Low Early Uptake of COVID-19 Vaccine in Hospital-Based Healthcare Workers, Georgia, March–July 2021. *Vaccines*, *10*(8), 1197. https://doi.org/10.3390/vaccines10081197

Martin, C. A., Marshall, C., Patel, P., Goss, C., Jenkins, D. R., Ellwood, C., Barton, L., Price, A., Brunskill, N. J., Khunti, K., & Pareek, M. (2021). SARS-CoV-2 vaccine uptake in a multi-ethnic UK healthcare workforce: A cross-sectional study. *PLOS Medicine*, *18*(11), e1003823. https://doi.org/10.1371/journal.pmed.1003823

Moucheraud, C., Phiri, K., Whitehead, H. S., Songo, J., Lungu, E., Chikuse, E., Phiri, S., van Oosterhout, J. J., & Hoffman, R. M. (2022). Uptake of the COVID-19 vaccine among healthcare workers in Malawi. *International Health*, ihac007. https://doi.org/10.1093/inthealth/ihac007

Narayan, P., Ts, S. K., Bv, M. M., Ghorai, P. A., Rupert, E., & Shetty, D. P. (2022). Uptake and impact of vaccination against COVID-19 among healthcare workers-evidence from a multicentre study. *American Journal of Infection Control*, *50*(3), 361–363. https://doi.org/10.1016/j.ajic.2021.10.036

Oliver, K., Raut, A., Pierre, S., Silvera, L., Boulos, A., Gale, A., Baum, A., Chory, A., Davis, N. J., D'Souza, D., Freeman, A., Goytia, C., Hamilton, A., Horowitz, C., Islam, N., Jeavons, J., Knudsen, J., Li, S., Lupi, J., … Maru, D. (2022). Factors associated with COVID-19 vaccine receipt at two integrated healthcare systems in New York City: A cross-sectional study of healthcare workers. *BMJ Open*, *12*(1), e053641. https://doi.org/10.1136/bmjopen-2021-053641

Rikitu Terefa, D., Shama, A. T., Feyisa, B. R., Ewunetu Desisa, A., Geta, E. T., Chego Cheme, M., & Tamiru Edosa, A. (2021). COVID-19 Vaccine Uptake and Associated Factors Among Health Professionals in Ethiopia. *Infection and Drug Resistance*, *14*, 5531–5541. https://doi.org/10.2147/IDR.S344647

Schrading, W. A., Trent, S. A., Paxton, J. H., Rodriguez, R. M., Swanson, M. B., Mohr, N. M., Talan, D. A., Project COVERED Emergency Department Network, Bahamon, M., Carlson, J. N., Chisolm-Straker, M., Driver, B., Faine, B., Galbraith, J., Giordano, P. A., Haran, J. P., Higgins, A., Hinson, J., House, S., … Weber, K. D. (2021). Vaccination rates and acceptance of SARS-CoV-2 vaccination among U.S. emergency department health care personnel. *Academic Emergency Medicine*, *28*(4), 455–458. https://doi.org/10.1111/acem.14236

Xu, B., Gao, X., Zhang, X., Hu, Y., Yang, H., & Zhou, Y.-H. (2021). Real-World Acceptance of COVID-19 Vaccines among Healthcare Workers in Perinatal Medicine in China. *Vaccines*, *9*(7), 704. https://doi.org/10.3390/vaccines9070704

Zdravkovic, M., Popadic, V., Nikolic, V., Klasnja, S., Brajkovic, M., Manojlovic, A., Nikolic, N., & Markovic-Denic, L. (2022). COVID-19 Vaccination Willingness and Vaccine Uptake among Healthcare Workers: A Single-Center Experience. *Vaccines*, *10*(4), 500. https://doi.org/10.3390/vaccines10040500
