# Supplementary material for: COVID-19 Vaccine Uptake among Healthcare Workers: A Systematic Review and Meta-Analysis
Source: Vaccines (Basel). 2022 Sep 29;10(10):1637. doi: 10.3390/vaccines10101637 (PMC9610263; doi:10.3390/vaccines10101637)
Supplement: Supplementary file 1 [file vaccines-10-01637-s001.zip › Supplementary Table S2.pdf]

Supplement Table S2. Quality of studies included in this systematic review.

	(Choi et al., 2022)	(Halbrook et al., 2022)	(Laiyemo et al., 2022)	(Lucaccioni et al., 2022)	(Dahie et al., 2022)	(Zdravkovic et al., 2022)
1. Were the criteria for inclusion in the sample clearly defined?	Yes	Yes	Yes	Yes	Yes	Yes
2. Were the study subjects and the setting described in detail?	Yes	Yes	Yes	Yes	Yes	Yes
3. Was the exposure measured in a valid and reliable way?	Yes	NA	Yes	Yes	Yes	Yes
4. Were objective, standard criteria used for measurement of the condition?	Yes	Yes	Yes	Yes	Yes	Yes
5. Were confounding factors identified?	Yes	NA	Yes	Yes	Yes	Yes
6. Were strategies to deal with confounding factors stated?	Yes	NA	Yes	Yes	Yes	Yes
7. Were the outcomes measured in a valid and reliable way?	Yes	Yes	Yes	Yes	Yes	Yes
8. Was appropriate statistical analysis used?	Yes	Yes	Yes	Yes	Yes	Yes
**Risk of bias**	**Low**	**Low**	**Low**	**Low**	**Low**	**Low**

NA: not applicable

Supplement Table S2 (continued). Quality of studies included in this systematic review.

	(Agha et al., 2021)	(Baniak et al., 2021)	(Xu et al., 2021)	(Martin et al., 2021)	(Narayan et al., 2022)	(Farah et al., 2022)	(Alya et al., 2022)	(Galanis et al., 2022)
1. Were the criteria for inclusion in the sample clearly defined?	Yes	Yes	Yes	Yes	No	Yes	Yes	Yes
2. Were the study subjects and the setting described in detail?	Yes	Yes	Yes	Yes	No	Yes	Yes	Yes
3. Was the exposure measured in a valid and reliable way?	Yes	Yes	Yes	NA	NA	Yes	Yes	Yes
4. Were objective, standard criteria used for measurement of the condition?	Yes	Yes	Yes	Yes	Yes	Yes	Yes	Yes
5. Were confounding factors identified?	Yes	Yes	Yes	NA	NA	Yes	Yes	Yes
6. Were strategies to deal with confounding factors stated?	Yes	Yes	Yes	NA	NA	Yes	Yes	Yes
7. Were the outcomes measured in a valid and reliable way?	Yes	Yes	Yes	Yes	Yes	Yes	Yes	Yes
8. Was appropriate statistical analysis used?	Yes	Yes	Yes	Yes	Yes	Yes	Yes	Yes
**Risk of bias**	**Low**	**Low**	**Low**	**Low**	**Moderate**	**Low**	**Low**	**Low**

NA: not applicable

Supplement Table S2 (continued). Quality of studies included in this systematic review.

	(Doran et al., 2022)	(Dubov et al., 2022)	(Rikitu Terefa et al., 2021)	(Oliver et al., 2022)	(Bedston et al., 2022)	(Moucheraud et al., 2022)	(Schrading et al., 2021)
1. Were the criteria for inclusion in the sample clearly defined?	Yes	Yes	Yes	Yes	Yes	Yes	Yes
2. Were the study subjects and the setting described in detail?	Yes	Yes	Yes	Yes	Yes	Yes	Yes
3. Was the exposure measured in a valid and reliable way?	Yes	Yes	Yes	Yes	Yes	Yes	NA
4. Were objective, standard criteria used for measurement of the condition?	Yes	Yes	Yes	Yes	Yes	Yes	Yes
5. Were confounding factors identified?	Yes	Yes	Yes	Yes	Yes	Yes	NA
6. Were strategies to deal with confounding factors stated?	Yes	Yes	Yes	Yes	Yes	Yes	NA
7. Were the outcomes measured in a valid and reliable way?	Yes	Yes	Yes	Yes	Yes	Yes	Yes
8. Was appropriate statistical analysis used?	Yes	Yes	Yes	Yes	Yes	Yes	Yes
**Risk of bias**	**Low**	**Low**	**Low**	**Low**	**Low**	**Low**	**Low**

NA: not applicable

Supplement Table S2 (continued). Quality of studies included in this systematic review.

	(Kraft et al., 2021)	(Abubakar et al., 2022)	(Gopaul et al., 2022)	(Akech et al., 2022)
1. Were the criteria for inclusion in the sample clearly defined?	Yes	No	Yes	Yes
2. Were the study subjects and the setting described in detail?	Yes	Yes	Yes	Yes
3. Was the exposure measured in a valid and reliable way?	NA	Yes	Yes	Yes
4. Were objective, standard criteria used for measurement of the condition?	Yes	Yes	Yes	Yes
5. Were confounding factors identified?	NA	Yes	Yes	Yes
6. Were strategies to deal with confounding factors stated?	NA	Yes	Yes	Yes
7. Were the outcomes measured in a valid and reliable way?	Yes	Yes	Yes	Yes
8. Was appropriate statistical analysis used?	Yes	Yes	Yes	Yes
**Risk of bias**	**Low**	**Low**	**Low**	**Low**

NA: not applicable

References

Abubakar, A. T., Suleiman, K., Ahmad, S. I., Suleiman, S. Y., Ibrahim, U. B., Suleiman, B. A., Haladu, S. A., Al-Mustapha, A. I., & Abubakar, M. I. (2022). *Acceptance of COVID-19 vaccine among healthcare workers in Katsina state, Northwest Nigeria* [Preprint]. Public and Global Health. https://doi.org/10.1101/2022.03.20.22272677

Agha, S., Chine, A., Lalika, M., Pandey, S., Seth, A., Wiyeh, A., Seng, A., Rao, N., & Badshah, A. (2021). Drivers of COVID-19 Vaccine Uptake amongst Healthcare Workers (HCWs) in Nigeria. *Vaccines*, *9*(10), 1162. https://doi.org/10.3390/vaccines9101162

Akech, G. M., Kanyike, A. M., Nassozi, A. G., Aguti, B., Nakawuki, A. W., Kimbugwe, D., Kiggundu, J., Maiteki, R., Mukyala, D., Bongomin, F., Obakiro, S. B., Rebecca, N., & Iramiot, J. S. (2022). *COVID-19 Vaccination Uptake and Self-Reported Side Effects among Healthcare Workers in Mbale City Eastern Uganda* [Preprint]. Infectious Diseases (except HIV/AIDS). https://doi.org/10.1101/2022.07.11.22277490

Alya, W. A., Maraqa, B., Nazzal, Z., Odeh, M., Makhalfa, R., Nassif, A., & Aabed, M. (2022). COVID-19 vaccine uptake and its associated factors among Palestinian healthcare workers: Expectations beaten by reality. *Vaccine*, *40*(26), 3713–3719. https://doi.org/10.1016/j.vaccine.2022.05.026

Baniak, L. M., Luyster, F. S., Raible, C. A., McCray, E. E., & Strollo, P. J. (2021). COVID-19 Vaccine Hesitancy and Uptake among Nursing Staff during an Active Vaccine Rollout. *Vaccines*, *9*(8), 858. https://doi.org/10.3390/vaccines9080858

Bedston, S., Akbari, A., Jarvis, C. I., Lowthian, E., Torabi, F., North, L., Lyons, J., Perry, M., Griffiths, L. J., Owen, R. K., Beggs, J., Chuter, A., Bradley, D. T., de Lusignan, S., Fry, R., Richard Hobbs, F. D., Hollinghurst, J., Katikireddi, S. V., Murphy, S., … Lyons, R. A. (2022). COVID-19 vaccine uptake, effectiveness, and waning in 82,959 health care workers: A national prospective cohort study in Wales. *Vaccine*, *40*(8), 1180–1189. https://doi.org/10.1016/j.vaccine.2021.11.061

Choi, K., Rondinelli, J., Cuenca, E., Lewin, B., Chang, J., Luo, Y. X., Bronstein, D., & Bruxvoort, K. (2022). Race/Ethnicity Differences in COVID-19 Vaccine Uptake Among Nurses. *Journal of Transcultural Nursing*, *33*(2), 134–140. https://doi.org/10.1177/10436596211065395

Dahie, H. A., Mohamoud, J. H., Adam, M. H., Garba, B., Dirie, N. I., Sh. Nur, M. A., & Mohamed, F. Y. (2022). COVID-19 Vaccine Coverage and Potential Drivers of Vaccine Uptake among Healthcare Workers in SOMALIA: A Cross-Sectional Study. *Vaccines*, *10*(7), 1116. https://doi.org/10.3390/vaccines10071116

Doran, J., Seyidov, N., Mehdiyev, S., Gon, G., Kissling, E., Herdman, T., Suleymanova, J., Rehse, A. P. C., Pebody, R., Katz, M. A., & Hagverdiyev, G. (2022). Factors associated with early uptake of COVID-19 vaccination among healthcare workers in Azerbaijan, 2021. *Influenza and Other Respiratory Viruses*, *16*(4), 626–631. https://doi.org/10.1111/irv.12978

Dubov, A., Distelberg, B. J., Abdul-Mutakabbir, J. C., Peteet, B., Roberts, L., Montgomery, S. B., Rockwood, N., Patel, P., Shoptaw, S., & Chrissian, A. A. (2022). Racial/Ethnic Variances in COVID-19 Inoculation among Southern California Healthcare Workers. *Vaccines*, *10*(8), 1331. https://doi.org/10.3390/vaccines10081331

Farah, W., Breeher, L., Shah, V., Hainy, C., Tommaso, C. P., & Swift, M. D. (2022). Disparities in COVID-19 vaccine uptake among health care workers. *Vaccine*, *40*(19), 2749–2754. https://doi.org/10.1016/j.vaccine.2022.03.045

Galanis, P., Moisoglou, I., Vraka, I., Siskou, O., Konstantakopoulou, O., Katsiroumpa, A., & Kaitelidou, D. (2022). Predictors of COVID-19 Vaccine Uptake in Healthcare Workers: A Cross-Sectional Study in Greece. *Journal of Occupational & Environmental Medicine*, *64*(4), e191–e196. https://doi.org/10.1097/JOM.0000000000002463

Gopaul, C. D., Ventour, D., & Thomas, D. (2022). *COVID-19 Vaccine Acceptance and Uptake Among Healthcare Workers in Trinidad & Tobago* [Preprint]. Public and Global Health. https://doi.org/10.1101/2022.05.09.22274854

Halbrook, M., Gadoth, A., Martin-Blais, R., Gray, A. N., Kashani, S., Kazan, C., Kane, B., Tobin, N. H., Ferbas, K. G., Aldrovandi, G. M., & Rimoin, A. W. (2022). Longitudinal Assessment of Coronavirus Disease 2019 Vaccine Acceptance and Uptake Among Frontline Medical Workers in Los Angeles, California. *Clinical Infectious Diseases*, *74*(7), 1166–1173. https://doi.org/10.1093/cid/ciab614

Kraft, K. B., Elgersma, I., Lyngstad, T. M., Elstrøm, P., & Telle, K. (2021). *COVID-19 vaccination rates among health care workers by immigrant background. A nation-wide registry study from Norway* [Preprint]. Public and Global Health. https://doi.org/10.1101/2021.09.17.21263619

Laiyemo, A. O., Asemota, J., Deonarine, A., Aduli, F., & McDonald-Pinkett, S. (2022). Minority Healthcare Workers' Perception of Safety and COVID-19 Vaccination Uptake. *Journal of General Internal Medicine*, *37*(4), 1006–1007. https://doi.org/10.1007/s11606-021-07299-y

Lucaccioni, H., Chakhunashvili, G., McKnight, C. J., Zardiashvili, T., Jorgensen, P., Pebody, R., Kissling, E., Katz, M. A., & Sanodze, L. (2022). Sociodemographic and Occupational Factors Associated with Low Early Uptake of COVID-19 Vaccine in Hospital-Based Healthcare Workers, Georgia, March–July 2021. *Vaccines*, *10*(8), 1197. https://doi.org/10.3390/vaccines10081197

Martin, C. A., Marshall, C., Patel, P., Goss, C., Jenkins, D. R., Ellwood, C., Barton, L., Price, A., Brunskill, N. J., Khunti, K., & Pareek, M. (2021). SARS-CoV-2 vaccine uptake in a multi-ethnic UK healthcare workforce: A cross-sectional study. *PLOS Medicine*, *18*(11), e1003823. https://doi.org/10.1371/journal.pmed.1003823

Moucheraud, C., Phiri, K., Whitehead, H. S., Songo, J., Lungu, E., Chikuse, E., Phiri, S., van Oosterhout, J. J., & Hoffman, R. M. (2022). Uptake of the COVID-19 vaccine among healthcare workers in Malawi. *International Health*, ihac007. https://doi.org/10.1093/inthealth/ihac007

Narayan, P., Ts, S. K., Bv, M. M., Ghorai, P. A., Rupert, E., & Shetty, D. P. (2022). Uptake and impact of vaccination against COVID-19 among healthcare workers-evidence from a multicentre study. *American Journal of Infection Control*, *50*(3), 361–363. https://doi.org/10.1016/j.ajic.2021.10.036

Oliver, K., Raut, A., Pierre, S., Silvera, L., Boulos, A., Gale, A., Baum, A., Chory, A., Davis, N. J., D'Souza, D., Freeman, A., Goytia, C., Hamilton, A., Horowitz, C., Islam, N., Jeavons, J., Knudsen, J., Li, S., Lupi, J., … Maru, D. (2022). Factors associated with COVID-19 vaccine receipt at two integrated healthcare systems in New York City: A cross-sectional study of healthcare workers. *BMJ Open*, *12*(1), e053641. https://doi.org/10.1136/bmjopen-2021-053641

Rikitu Terefa, D., Shama, A. T., Feyisa, B. R., Ewunetu Desisa, A., Geta, E. T., Chego Cheme, M., & Tamiru Edosa, A. (2021). COVID-19 Vaccine Uptake and Associated Factors Among Health Professionals in Ethiopia. *Infection and Drug Resistance*, *14*, 5531–5541. https://doi.org/10.2147/IDR.S344647

Schrading, W. A., Trent, S. A., Paxton, J. H., Rodriguez, R. M., Swanson, M. B., Mohr, N. M., Talan, D. A., Project COVERED Emergency Department Network, Bahamon, M., Carlson, J. N., Chisolm-Straker, M., Driver, B., Faine, B., Galbraith, J., Giordano, P. A., Haran, J. P., Higgins, A., Hinson, J., House, S., … Weber, K. D. (2021). Vaccination rates and acceptance of SARS-CoV-2 vaccination among U.S. emergency department health care personnel. *Academic Emergency Medicine*, *28*(4), 455–458. https://doi.org/10.1111/acem.14236

Xu, B., Gao, X., Zhang, X., Hu, Y., Yang, H., & Zhou, Y.-H. (2021). Real-World Acceptance of COVID-19 Vaccines among Healthcare Workers in Perinatal Medicine in China. *Vaccines*, *9*(7), 704. https://doi.org/10.3390/vaccines9070704

Zdravkovic, M., Popadic, V., Nikolic, V., Klasnja, S., Brajkovic, M., Manojlovic, A., Nikolic, N., & Markovic-Denic, L. (2022). COVID-19 Vaccination Willingness and Vaccine Uptake among Healthcare Workers: A Single-Center Experience. *Vaccines*, *10*(4), 500. https://doi.org/10.3390/vaccines10040500
