# Supplementary material for: COVID-19 Vaccine Uptake among Healthcare Workers: A Systematic Review and Meta-Analysis
Source: Vaccines (Basel). 2022 Sep 29;10(10):1637. doi: 10.3390/vaccines10101637 (PMC9610263; doi:10.3390/vaccines10101637)
Supplement: Supplementary file 1 [file vaccines-10-01637-s001.zip › Supplementary Table S3.pdf]

Supplementary Table S3. COVID-19 vaccination uptake among healthcare workers.

**Reference**	**All healthcare workers**			**Physicians**			**Nurses**		
	**Total number**	**Number of vaccinated**	**Vaccination uptake (%)**	**Total number**	**Number of vaccinated**	**Vaccination uptake (%)**	**Total number**	**Number of vaccinated**	**Vaccination uptake (%)**
(Choi et al., 2022)	1183	980	82.8				1183	980	82.8
(Halbrook et al., 2022)	400	384	96.0						
(Laiyemo et al., 2022)	269	207	77.0	62	58	93.6	78	62	79.5
(Lucaccioni et al., 2022)	1533	274	17.9	314	130	41.4			
(Dahie et al., 2022)	1281	623	48.6						
(Zdravkovic et al., 2022)	575	383	66.6	130	96	73.8	277	171	61.7
(Agha et al., 2021)	496	164	33.1	79	40	50.6			
(Baniak et al., 2021)	276	230	83.3						
(Xu et al., 2021)	1051	906	86.2	670	582	64.2			

(Martin et al., 2021)	19,044	12,278	64.5	3001	1721	57.3			
(Narayan et al., 2022)	14,837	13.335	90.0						
(Farah et al., 2022)	65,270	51,320	78.6	6628	6351	95.8	16,030	12,644	78.9
(Alya et al., 2022)	1018	677	66.5	438	323	73.7	292	182	62.3
(Galanis et al., 2022)	885	810	91.5	220	207	94.1	396	365	92.2
(Doran et al., 2022)	1575	1133	73.0	408	275	67.4			
(Dubov et al., 2022)	1131	950	84.0						
(Rikitu Terefa et al., 2021)	522	324	62.1	30	27	90.0	124	67	54.0
(Oliver et al., 2022)	1933	1775	91.8	268	254	94.8	265	192	72.5
(Bedston et al., 2022)	82,959	74,489	89.8						
(Moucheraud et al., 2022)	400	330	82.5						
(Schrading et al., 2021)	1321	1136	86.0	674	637	94.5	345	268	77.7
(Kraft et al., 2021)	356,053	327,569	92.0	22,432	21,759	97.0	58,745	54,633	93.0

(Abubakar et al., 2022)	793	714	90.0						
(Gopaul et al., 2022)	584	514	88.0	270	262	97.0	220	181	82.3
(Akech et al., 2022)	172	119	69.0						

References

Abubakar, A. T., Suleiman, K., Ahmad, S. I., Suleiman, S. Y., Ibrahim, U. B., Suleiman, B. A., Haladu, S. A., Al-Mustapha, A. I., & Abubakar, M. I. (2022). *Acceptance of COVID-19 vaccine among healthcare workers in Katsina state, Northwest Nigeria* [Preprint]. Public and Global Health. https://doi.org/10.1101/2022.03.20.22272677

Agha, S., Chine, A., Lalika, M., Pandey, S., Seth, A., Wiyeh, A., Seng, A., Rao, N., & Badshah, A. (2021). Drivers of COVID-19 Vaccine Uptake amongst Healthcare Workers (HCWs) in Nigeria. *Vaccines*, *9*(10), 1162. https://doi.org/10.3390/vaccines9101162

Akech, G. M., Kanyike, A. M., Nassozi, A. G., Aguti, B., Nakawuki, A. W., Kimbugwe, D., Kiggundu, J., Maiteki, R., Mukyala, D., Bongomin, F., Obakiro, S. B., Rebecca, N., & Iramiot, J. S. (2022). *COVID-19 Vaccination Uptake and Self-Reported Side Effects among Healthcare Workers in Mbale City Eastern Uganda* [Preprint]. Infectious Diseases (except HIV/AIDS). https://doi.org/10.1101/2022.07.11.22277490

Alya, W. A., Maraqa, B., Nazzal, Z., Odeh, M., Makhalfa, R., Nassif, A., & Aabed, M. (2022). COVID-19 vaccine uptake and its associated factors among Palestinian healthcare workers: Expectations beaten by reality. *Vaccine*, *40*(26), 3713–3719. https://doi.org/10.1016/j.vaccine.2022.05.026

Baniak, L. M., Luyster, F. S., Raible, C. A., McCray, E. E., & Strollo, P. J. (2021). COVID-19 Vaccine Hesitancy and Uptake among Nursing Staff during an Active Vaccine Rollout. *Vaccines*, *9*(8), 858. https://doi.org/10.3390/vaccines9080858

Bedston, S., Akbari, A., Jarvis, C. I., Lowthian, E., Torabi, F., North, L., Lyons, J., Perry, M., Griffiths, L. J., Owen, R. K., Beggs, J., Chuter, A., Bradley, D. T., de Lusignan, S., Fry, R., Richard Hobbs, F. D., Hollinghurst, J., Katikireddi, S. V., Murphy, S., … Lyons, R. A. (2022). COVID-19 vaccine uptake, effectiveness, and waning in 82,959 health care workers: A national prospective cohort study in Wales. *Vaccine*, *40*(8), 1180–1189. https://doi.org/10.1016/j.vaccine.2021.11.061

Choi, K., Rondinelli, J., Cuenca, E., Lewin, B., Chang, J., Luo, Y. X., Bronstein, D., & Bruxvoort, K. (2022). Race/Ethnicity Differences in COVID-19 Vaccine Uptake Among Nurses. *Journal of Transcultural Nursing*, *33*(2), 134–140. https://doi.org/10.1177/10436596211065395

Dahie, H. A., Mohamoud, J. H., Adam, M. H., Garba, B., Dirie, N. I., Sh. Nur, M. A., & Mohamed, F. Y. (2022). COVID-19 Vaccine Coverage and Potential Drivers of Vaccine Uptake among Healthcare Workers in SOMALIA: A Cross-Sectional Study. *Vaccines*, *10*(7), 1116. https://doi.org/10.3390/vaccines10071116

Doran, J., Seyidov, N., Mehdiyev, S., Gon, G., Kissling, E., Herdman, T., Suleymanova, J., Rehse, A. P. C., Pebody, R., Katz, M. A., & Hagverdiyev, G. (2022). Factors associated with early uptake of COVID-19 vaccination among healthcare workers in Azerbaijan, 2021. *Influenza and Other Respiratory Viruses*, *16*(4), 626–631. https://doi.org/10.1111/irv.12978

Dubov, A., Distelberg, B. J., Abdul-Mutakabbir, J. C., Peteet, B., Roberts, L., Montgomery, S. B., Rockwood, N., Patel, P., Shoptaw, S., & Chrissian, A. A. (2022). Racial/Ethnic Variances in COVID-19 Inoculation among Southern California Healthcare Workers. *Vaccines*, *10*(8), 1331. https://doi.org/10.3390/vaccines10081331

Farah, W., Breeher, L., Shah, V., Hainy, C., Tommaso, C. P., & Swift, M. D. (2022). Disparities in COVID-19 vaccine uptake among health care workers. *Vaccine*, *40*(19), 2749–2754. https://doi.org/10.1016/j.vaccine.2022.03.045

Galanis, P., Moisoglou, I., Vraka, I., Siskou, O., Konstantakopoulou, O., Katsiroumpa, A., & Kaitelidou, D. (2022). Predictors of COVID-19 Vaccine Uptake in Healthcare Workers: A Cross-Sectional Study in Greece. *Journal of Occupational & Environmental Medicine*, *64*(4), e191–e196. https://doi.org/10.1097/JOM.0000000000002463

Gopaul, C. D., Ventour, D., & Thomas, D. (2022). *COVID-19 Vaccine Acceptance and Uptake Among Healthcare Workers in Trinidad & Tobago* [Preprint]. Public and Global Health. https://doi.org/10.1101/2022.05.09.22274854

Halbrook, M., Gadoth, A., Martin-Blais, R., Gray, A. N., Kashani, S., Kazan, C., Kane, B., Tobin, N. H., Ferbas, K. G., Aldrovandi, G. M., & Rimoin, A. W. (2022). Longitudinal Assessment of Coronavirus Disease 2019 Vaccine Acceptance and Uptake Among Frontline Medical Workers in Los Angeles, California. *Clinical Infectious Diseases*, *74*(7), 1166–1173. https://doi.org/10.1093/cid/ciab614

Kraft, K. B., Elgersma, I., Lyngstad, T. M., Elstrøm, P., & Telle, K. (2021). *COVID-19 vaccination rates among health care workers by immigrant background. A nation-wide registry study from Norway* [Preprint]. Public and Global Health. https://doi.org/10.1101/2021.09.17.21263619

Laiyemo, A. O., Asemota, J., Deonarine, A., Aduli, F., & McDonald-Pinkett, S. (2022). Minority Healthcare Workers' Perception of Safety and COVID-19 Vaccination Uptake. *Journal of General Internal Medicine*, *37*(4), 1006–1007. https://doi.org/10.1007/s11606-021-07299-y

Lucaccioni, H., Chakhunashvili, G., McKnight, C. J., Zardiashvili, T., Jorgensen, P., Pebody, R., Kissling, E., Katz, M. A., & Sanodze, L. (2022). Sociodemographic and Occupational Factors Associated with Low Early Uptake of COVID-19 Vaccine in Hospital-Based Healthcare Workers, Georgia, March–July 2021. *Vaccines*, *10*(8), 1197. https://doi.org/10.3390/vaccines10081197

Martin, C. A., Marshall, C., Patel, P., Goss, C., Jenkins, D. R., Ellwood, C., Barton, L., Price, A., Brunskill, N. J., Khunti, K., & Pareek, M. (2021). SARS-CoV-2 vaccine uptake in a multi-ethnic UK healthcare workforce: A cross-sectional study. *PLOS Medicine*, *18*(11), e1003823. https://doi.org/10.1371/journal.pmed.1003823

Moucheraud, C., Phiri, K., Whitehead, H. S., Songo, J., Lungu, E., Chikuse, E., Phiri, S., van Oosterhout, J. J., & Hoffman, R. M. (2022). Uptake of the COVID-19 vaccine among healthcare workers in Malawi. *International Health*, ihac007. https://doi.org/10.1093/inthealth/ihac007

Narayan, P., Ts, S. K., Bv, M. M., Ghorai, P. A., Rupert, E., & Shetty, D. P. (2022). Uptake and impact of vaccination against COVID-19 among healthcare workers-evidence from a multicentre study. *American Journal of Infection Control*, *50*(3), 361–363. https://doi.org/10.1016/j.ajic.2021.10.036

Oliver, K., Raut, A., Pierre, S., Silvera, L., Boulos, A., Gale, A., Baum, A., Chory, A., Davis, N. J., D'Souza, D., Freeman, A., Goytia, C., Hamilton, A., Horowitz, C., Islam, N., Jeavons, J., Knudsen, J., Li, S., Lupi, J., … Maru, D. (2022). Factors associated with COVID-19 vaccine receipt at two integrated healthcare systems in New York City: A cross-sectional study of healthcare workers. *BMJ Open*, *12*(1), e053641. https://doi.org/10.1136/bmjopen-2021-053641

Rikitu Terefa, D., Shama, A. T., Feyisa, B. R., Ewunetu Desisa, A., Geta, E. T., Chego Cheme, M., & Tamiru Edosa, A. (2021). COVID-19 Vaccine Uptake and Associated Factors Among Health Professionals in Ethiopia. *Infection and Drug Resistance*, *14*, 5531–5541. https://doi.org/10.2147/IDR.S344647

Schrading, W. A., Trent, S. A., Paxton, J. H., Rodriguez, R. M., Swanson, M. B., Mohr, N. M., Talan, D. A., Project COVERED Emergency Department Network, Bahamon, M., Carlson, J. N., Chisolm-Straker, M., Driver, B., Faine, B., Galbraith, J., Giordano, P. A., Haran, J. P., Higgins, A., Hinson, J., House, S., … Weber, K. D. (2021). Vaccination rates and acceptance of SARS-CoV-2 vaccination among U.S. emergency department health care personnel. *Academic Emergency Medicine*, *28*(4), 455–458. https://doi.org/10.1111/acem.14236

Xu, B., Gao, X., Zhang, X., Hu, Y., Yang, H., & Zhou, Y.-H. (2021). Real-World Acceptance of COVID-19 Vaccines among Healthcare Workers in Perinatal Medicine in China. *Vaccines*, *9*(7), 704. https://doi.org/10.3390/vaccines9070704

Zdravkovic, M., Popadic, V., Nikolic, V., Klasnja, S., Brajkovic, M., Manojlovic, A., Nikolic, N., & Markovic-Denic, L. (2022). COVID-19 Vaccination Willingness and Vaccine Uptake among Healthcare Workers: A Single-Center Experience. *Vaccines*, *10*(4), 500. https://doi.org/10.3390/vaccines10040500
