# Supplementary material for: COVID-19 Vaccine Uptake among Healthcare Workers: A Systematic Review and Meta-Analysis
Source: Vaccines (Basel). 2022 Sep 29;10(10):1637. doi: 10.3390/vaccines10101637 (PMC9610263; doi:10.3390/vaccines10101637)
Supplement: Supplementary file 1 [file vaccines-10-01637-s001.zip › Supplementary Table S4.pdf]

Supplementary Table S4. Measures of effect and precision between predictors and COVID-19 vaccine uptake among healthcare workers.

Reference	Older age	Males	Married	Higher educational level	White race	Higher income	Physicians	Work experience	Chronic condition
(Choi et al., 2022)	-	-	-	-	East Asian vs. white: 1.14 (95% CI: 1.06-1.24); Filipino vs. white: 1.14 (95% CI:1.08-1.20)	-	-	-	-
(Laiyemo et al., 2022)	≥40 years vs. <40 years: 2.90 (95% CI: 1.24-6.81)	NS	NS	College graduate vs. less than college: 4.87 (95% CI: 1.86-12.73)	NS	-	NS	-	-
(Lucaccioni et al., 2022)	30-39 years vs. 18-29: 1.22 (95% CI: 0.76-1.97); 40-49 years vs. 18-29: 1.83 (95% CI: 1.14-2.94); 50-59 years vs. 18-29: 2.40 (95% CI: 1.50-3.88); ≥60 years vs. 18-29: 1.90 (95% CI: 1.10-3.27)	NS	-	-	-	-	Nurses and midwives vs. physicians: 0.22 (95% CI: 0.15-0.32); Ancillary workers vs. physicians: 0.08 (95% CI: 0.04-0.15); Other healthcare works vs. physicians: 0.15 (95% CI: 0.07-0.30)	-	NS
(Dahie et al., 2022)	36-45 years vs. ≤25: 2.4 (95% CI: 1.29-4.44)	2.2 (95% CI: 1.70-2.75)	-	Bachelor degree vs. certificate: 1.90 (95% CI: 1.11-3.49); Postgraduate degree vs. certificate: 4.10 (95% CI: 2.13-8.25)	-	-	Physicians vs. midwives: 1.80 (95% CI: 1.17-2.83)	-	NS
(Zdravkovic et al., 2022)	30-39 years vs. 18-29: 2.09 (95% CI: 1.08-4.05); 40-49 years vs. 18-29: 3.92 (95% CI: 1.97-7.83); 50-59 years vs. 18-29: 4.69 (95% CI: 2.27-9.71)	Females vs. males: 1.92 (95% CI: 1.04-3.55)	-	-	-	-	Physicians vs. nurses: 2.56 (95% CI: 1.33-5.00)	NS	-
(Agha et al., 2021)	-	-	-	Bachelor degree vs. certificate: 2.60 (95% CI: 1.21-5.59); Diploma vs. certificate: 2.82 (95% CI: 1.30-6.12); Master's or higher vs. certificate: 2.40 (95% CI: 0.95-6.07)	-	-	Physicians vs. nurses: 3.08 (95% CI: 1.41-6.77)	-	-
(Baniak et	NS	NS	NS	-	NS	-	-	NS	-

al., 2021)									
(Xu et al., 2021)	NS	NS	-	NS	-	-	NS	-	-
(Martin et al., 2021)	≤30 years vs. 41-50:0.48 (95% CI: 0.44-0.53); 31-40 years vs. 41-50: 0.64 (95% CI: 0.58-0.70); 51-60 years vs. 41-50: 1.19 (95% CI: 1.07-1.31); ≥61 years vs. 41-50: 1.18 (95% CI: 1.03-1.36)	1.24 (95% CI: 1.15-1.35)	-	-	South Asian vs. white: 0.67 (95% CI: 0.62-0.72); Black vs. white: 0.30 (95% CI: 0.26-0.34)	-	Allied health professionals vs. physicians: 1.40 (95% CI: 1.20-1.62); healthcare scientists vs. physicians: 1.69 (95% CI: 1.41-2.02)	-	-
(Farah et al., 2022)	25-34 years vs. <25: 1.07 (95% CI: 0.98-1.17); 35-44 years vs. <25: 1.45 (95% CI: 1.32-2.17); 45-54 years vs. <25: 1.95 (95% CI: 1.77-2.15); 55-64 years vs. <25: 2.76 (95% CI: 2.50-3.04); ≥65 years vs. <25: 3.95 (95% CI: 3.34-4.66)	1.20 (95% CI: 1.14-1.27)	-	-	Black vs. white: 0.55 (95% CI: 0.50-0.60); Hispanic vs. white: 1.15 (95% CI: 1.04-1.27); Asian vs. white: 1.74 (95% CI: 1.57-1.93)	-	Physicians vs. nonclinical support staff: 9.20 (95% CI: 8.06-10.51)	-	-
(Alya et al., 2022)	-	1.5 (95% CI: 1.1-2.1)	Unmarried vs. married: 1.30 (95% CI: 1.10-1.80)	-	-	1.90 (95% CI: 1.40-2.80)	-	-	-
(Galanis et al., 2022)	NS	Females vs. males: 3.36 (95% CI: 1.11-10.23)	NS	NS	-	NS	NS	NS	NS
(Doran et al., 2022)	35-49 years vs. <35: 1.62 (95% CI: 1.08-2.40); 50-59 years vs. <35: 2.18 (95% CI: 1.41-3.38); ≥60 years vs. <35: 1.80 (95% CI: 1.09-2.86)	NS	-	-	-	-	NS	-	NS
(Dubov et al., 2022)	0.63 (95% CI: 0.49-0.80)	-	-	-	-	-	-	-	-
(Rikitu Terefa et al., 2021)	40-49 years vs. 18-29: 12.97 (95% CI: 2.36-71.21); ≥50 years vs. 18-29: 18.95 (95% CI: 2.04-36.29)	2.91 (95% CI: 1.05-8.09)	-	NS	-	NS	NS	-	NS
(Oliver et al.,	NS	NS	-	-	Black vs.	-	Nurses vs. physicians:	-	-

2022)					white: 0.38 (95% CI: 0.24-0.59)		0.37 (95% CI: 0.21-0.65); allied and other health professionals vs. physicians: 0.48 (95% CI: 0.27-0.81)		
(Bedston et al., 2022)	30-39 years vs. ≤29: 1.08 (95% CI: 1.05-1.10); 40-49 years vs. ≤29: 1.38 (95% CI: 1.35-1.42); 50-59 years vs. ≤29: 1.60 (95% CI: 1.56-1.64); ≥60 years vs. ≤29: 1.65 (95% CI: 1.60-1.70)	NS	-	-	Others vs. white: 0.93 (95% CI: 0.90-0.96)	-	Physicians vs. nurses: 1.46 (95% CI: 1.41 to 1.52)	-	-
(Moucheraud et al., 2022)	NS	NS	-	-	-	-	NS	-	NS
(Abubakar et al., 2022)	30-39 years vs. ≤29: 7.06 (95% CI: 2.36-21.07); 40-49 years vs. ≤29: 5.05 (95% CI: 1.46-17.49)	NS	-	-	-	-	-	-	-
(Gopaul et al., 2022)	>35 years vs. ≤35: 12.5 (95% CI: 1.47-100)	NS	-	-	-	-	Physicians vs. nurses: 11.11 (95% CI: 1.72-100)	-	-
(Akech et al., 2022)	>41 years vs. ≤40: 2.70 (95% CI: 0.50-12.30)	-	-	-	-	-	NS	-	NS

Values express odds ratios. CI: confidence interval; NS: non-significant

References

Abubakar, A. T., Suleiman, K., Ahmad, S. I., Suleiman, S. Y., Ibrahim, U. B., Suleiman, B. A., Haladu, S. A., Al-Mustapha, A. I., & Abubakar, M. I. (2022). *Acceptance of COVID-19 vaccine among healthcare workers in Katsina state, Northwest Nigeria* [Preprint]. Public and Global Health. https://doi.org/10.1101/2022.03.20.22272677

Agha, S., Chine, A., Lalika, M., Pandey, S., Seth, A., Wiyeh, A., Seng, A., Rao, N., & Badshah, A. (2021). Drivers of COVID-19 Vaccine Uptake amongst Healthcare Workers (HCWs) in Nigeria. *Vaccines*, *9*(10), 1162. https://doi.org/10.3390/vaccines9101162

Akech, G. M., Kanyike, A. M., Nassozi, A. G., Aguti, B., Nakawuki, A. W., Kimbugwe, D., Kiggundu, J., Maiteki, R., Mukyala, D., Bongomin, F., Obakiro, S. B., Rebecca, N., & Iramiot, J. S. (2022). *COVID-19 Vaccination Uptake and Self-Reported Side Effects among Healthcare Workers in Mbale City Eastern Uganda* [Preprint]. Infectious Diseases (except HIV/AIDS). https://doi.org/10.1101/2022.07.11.22277490

Alya, W. A., Maraqa, B., Nazzal, Z., Odeh, M., Makhalfa, R., Nassif, A., & Aabed, M. (2022). COVID-19 vaccine uptake and its associated factors among Palestinian healthcare workers: Expectations beaten by reality. *Vaccine*, *40*(26), 3713–3719. https://doi.org/10.1016/j.vaccine.2022.05.026

Baniak, L. M., Luyster, F. S., Raible, C. A., McCray, E. E., & Strollo, P. J. (2021). COVID-19 Vaccine Hesitancy and Uptake among Nursing Staff during an Active Vaccine Rollout. *Vaccines*, *9*(8), 858. https://doi.org/10.3390/vaccines9080858

Bedston, S., Akbari, A., Jarvis, C. I., Lowthian, E., Torabi, F., North, L., Lyons, J., Perry, M., Griffiths, L. J., Owen, R. K., Beggs, J., Chuter, A., Bradley, D. T., de Lusignan, S., Fry, R., Richard Hobbs, F. D., Hollinghurst, J., Katikireddi, S. V., Murphy, S., … Lyons, R. A. (2022). COVID-19 vaccine uptake, effectiveness, and waning in 82,959 health care workers: A national prospective cohort study in Wales. *Vaccine*, *40*(8), 1180–1189. https://doi.org/10.1016/j.vaccine.2021.11.061

Choi, K., Rondinelli, J., Cuenca, E., Lewin, B., Chang, J., Luo, Y. X., Bronstein, D., & Bruxvoort, K. (2022). Race/Ethnicity Differences in COVID-19 Vaccine Uptake Among Nurses. *Journal of Transcultural Nursing*, *33*(2), 134–140. https://doi.org/10.1177/10436596211065395

Dahie, H. A., Mohamoud, J. H., Adam, M. H., Garba, B., Dirie, N. I., Sh. Nur, M. A., & Mohamed, F. Y. (2022). COVID-19 Vaccine Coverage and Potential Drivers of Vaccine Uptake among Healthcare Workers in SOMALIA: A Cross-Sectional Study. *Vaccines*, *10*(7), 1116. https://doi.org/10.3390/vaccines10071116

Doran, J., Seyidov, N., Mehdiyev, S., Gon, G., Kissling, E., Herdman, T., Suleymanova, J., Rehse, A. P. C., Pebody, R., Katz, M. A., & Hagverdiyev, G. (2022). Factors associated with early uptake of COVID-19 vaccination among healthcare workers in Azerbaijan, 2021. *Influenza and Other Respiratory Viruses*, *16*(4), 626–631. https://doi.org/10.1111/irv.12978

Dubov, A., Distelberg, B. J., Abdul-Mutakabbir, J. C., Peteet, B., Roberts, L., Montgomery, S. B., Rockwood, N., Patel, P., Shoptaw, S., & Chrissian, A. A. (2022). Racial/Ethnic Variances in COVID-19 Inoculation among Southern California Healthcare Workers. *Vaccines*, *10*(8), 1331. https://doi.org/10.3390/vaccines10081331

Farah, W., Breeher, L., Shah, V., Hainy, C., Tommaso, C. P., & Swift, M. D. (2022). Disparities in COVID-19 vaccine uptake among health care workers. *Vaccine*, *40*(19), 2749–2754. https://doi.org/10.1016/j.vaccine.2022.03.045

Galanis, P., Moisoglou, I., Vraka, I., Siskou, O., Konstantakopoulou, O., Katsiroumpa, A., & Kaitelidou, D. (2022). Predictors of COVID-19 Vaccine Uptake in Healthcare Workers: A Cross-Sectional Study in Greece. *Journal of Occupational & Environmental Medicine*, *64*(4), e191–e196. https://doi.org/10.1097/JOM.0000000000002463

Gopaul, C. D., Ventour, D., & Thomas, D. (2022). *COVID-19 Vaccine Acceptance and Uptake Among Healthcare Workers in Trinidad & Tobago* [Preprint]. Public and Global Health. https://doi.org/10.1101/2022.05.09.22274854

Laiyemo, A. O., Asemota, J., Deonarine, A., Aduli, F., & McDonald-Pinkett, S. (2022). Minority Healthcare Workers' Perception of Safety and COVID-19 Vaccination Uptake. *Journal of General Internal Medicine*, *37*(4), 1006–1007. https://doi.org/10.1007/s11606-021-07299-y

Lucaccioni, H., Chakhunashvili, G., McKnight, C. J., Zardiashvili, T., Jorgensen, P., Pebody, R., Kissling, E., Katz, M. A., & Sanodze, L. (2022). Sociodemographic and Occupational Factors Associated with Low Early Uptake of COVID-19 Vaccine in Hospital-Based Healthcare Workers, Georgia, March–July 2021. *Vaccines*, *10*(8), 1197. https://doi.org/10.3390/vaccines10081197

Martin, C. A., Marshall, C., Patel, P., Goss, C., Jenkins, D. R., Ellwood, C., Barton, L., Price, A., Brunskill, N. J., Khunti, K., & Pareek, M. (2021). SARS-CoV-2 vaccine uptake in a multi-ethnic UK healthcare workforce: A cross-sectional study. *PLOS Medicine*, *18*(11), e1003823. https://doi.org/10.1371/journal.pmed.1003823

Moucheraud, C., Phiri, K., Whitehead, H. S., Songo, J., Lungu, E., Chikuse, E., Phiri, S., van Oosterhout, J. J., & Hoffman, R. M. (2022). Uptake of the COVID-19 vaccine among healthcare workers in Malawi. *International Health*, ihac007. https://doi.org/10.1093/inthealth/ihac007

Oliver, K., Raut, A., Pierre, S., Silvera, L., Boulos, A., Gale, A., Baum, A., Chory, A., Davis, N. J., D'Souza, D., Freeman, A., Goytia, C., Hamilton, A., Horowitz, C., Islam, N., Jeavons, J., Knudsen, J., Li, S., Lupi, J., … Maru, D. (2022). Factors associated with

COVID-19 vaccine receipt at two integrated healthcare systems in New York City: A cross-sectional study of healthcare workers. *BMJ Open*, *12*(1), e053641. https://doi.org/10.1136/bmjopen-2021-053641

Rikitu Terefa, D., Shama, A. T., Feyisa, B. R., Ewunetu Desisa, A., Geta, E. T., Chego Cheme, M., & Tamiru Edosa, A. (2021). COVID-19 Vaccine Uptake and Associated Factors Among Health Professionals in Ethiopia. *Infection and Drug Resistance*, *14*, 5531–5541. https://doi.org/10.2147/IDR.S344647

Xu, B., Gao, X., Zhang, X., Hu, Y., Yang, H., & Zhou, Y.-H. (2021). Real-World Acceptance of COVID-19 Vaccines among Healthcare Workers in Perinatal Medicine in China. *Vaccines*, *9*(7), 704. https://doi.org/10.3390/vaccines9070704

Zdravkovic, M., Popadic, V., Nikolic, V., Klasnja, S., Brajkovic, M., Manojlovic, A., Nikolic, N., & Markovic-Denic, L. (2022). COVID-19 Vaccination Willingness and Vaccine Uptake among Healthcare Workers: A Single-Center Experience. *Vaccines*, *10*(4), 500. https://doi.org/10.3390/vaccines10040500
