# Supplementary material for: COVID-19 Vaccine Uptake among Healthcare Workers: A Systematic Review and Meta-Analysis
Source: Vaccines (Basel). 2022 Sep 29;10(10):1637. doi: 10.3390/vaccines10101637 (PMC9610263; doi:10.3390/vaccines10101637)
Supplement: Supplementary file 1 [file vaccines-10-01637-s001.zip › Supplementary Table S5.pdf]

Supplementary Table S5. Measures of effect between predictors and COVID-19 vaccine uptake among healthcare workers.

**Reference**	**History of SARS-CoV-2 infection**	**Higher severity perception of COVID-19**	**Direct COVID-19 patients care**	**Self-rated health status**	**Influenza vaccine**	**Confidence in COVID-19 vaccine effectiveness**	**Confidence in COVID-19 vaccine safety**	**Knowledge about COVID-19 vaccine**	**Information about COVID-19 vaccine**
(Lucaccioni et al., 2022)	0.57 (95% CI: 0.42-0.78)	-	NS	NS	2.98 (95% CI: 2.19-4.08)	10.90 (95% CI: 3.88-45.70)	-	-	NS
(Dahie et al., 2022)	-	-	2.00 (95% CI: 1.29-2.14)	-	-	-	-	-	-
(Zdravkovic et al., 2022)	NS	-	-	-	-	-	-	-	-
(Baniak et al., 2021)	-	-	-	-	-	NS	7.48 (95% CI: 4.41-12.69)	NS	NS
(Xu et al., 2021)	-	-	-	-	-	-	1.65 (95% CI: 1.04-2.62)	-	-
(Martin et al., 2021)	0.71 (95% CI: 0.60-0.85)	-	-	-	-	-	-	-	-
(Farah et al., 2022)	0.55 (95% CI: 0.51-0.59)	-	1.11 (95% CI: 1.04-1.22)	-	-	-	-	-	-
(Alya et al., 2022)	0.42 (0.30-0.59)	-	-	-	-	-	1.60 (95% CI: 1.40-1.70)	-	-
(Galanis et al., 2022)	NS	NS	NS	-	4.25 (95% CI: 1.86-9.75)	1.45 (95% CI: 1.18-1.78)	1.43 (95% CI: 1.18-1.72)	-	-
(Doran et al., 2022)	No vs. yes: 0.11 (95% CI:	NS	1.41 (95% CI: 1.10-	Good vs. excellent	2.30 (95% CI: 1.67-	NS	4.00 (95% CI:	NS	-

	0.08-0.15)		1.84)	or very good: 0.57 (95% CI: 0.37-0.85); fair or poor vs. excellent or very good: 0.49 (95% CI: 0.31-0.81)	3.20)		2.04-7.69)		
(Dubov et al., 2022)	0.17 (95% CI: 0.11-0.25)	-	-	-	-	-	-	-	-
(Rikitu Terefa et al., 2021)	-	-	2.38 (95% CI: 1.16-5.00)	NS	-	-	-	-	-
(Oliver et al., 2022)	-	-	-	-	3.57 (95% CI: 2.27-5.56)	-	2.56 (95% CI: 1.82-3.57)	-	-
(Abubakar et al., 2022)	NS	-	-	-	-	-	-	-	-
(Gopaul et al., 2022)	-	-	-	-	-	NS	-	-	-
(Akech et al., 2022)	-	14.3 (95% CI: 1.1-181.2)	NS	-	-	-	-	-	-

Values express odds ratios. CI: confidence interval; NS: non-significant

References

Abubakar, A. T., Suleiman, K., Ahmad, S. I., Suleiman, S. Y., Ibrahim, U. B., Suleiman, B. A., Haladu, S. A., Al-Mustapha, A. I., & Abubakar, M. I. (2022). *Acceptance of COVID-19 vaccine among healthcare workers in Katsina state, Northwest Nigeria* [Preprint]. Public and Global Health. https://doi.org/10.1101/2022.03.20.22272677

Akech, G. M., Kanyike, A. M., Nassozi, A. G., Aguti, B., Nakawuki, A. W., Kimbugwe, D., Kiggundu, J., Maiteki, R., Mukyala, D., Bongomin, F., Obakiro, S. B., Rebecca, N., & Iramiot, J. S. (2022). *COVID-19 Vaccination Uptake and Self-Reported Side Effects among Healthcare Workers in Mbale City Eastern Uganda* [Preprint]. Infectious Diseases (except HIV/AIDS). https://doi.org/10.1101/2022.07.11.22277490

Alya, W. A., Maraqa, B., Nazzal, Z., Odeh, M., Makhalfa, R., Nassif, A., & Aabed, M. (2022). COVID-19 vaccine uptake and its associated factors among Palestinian healthcare workers: Expectations beaten by reality. *Vaccine*, *40*(26), 3713–3719. https://doi.org/10.1016/j.vaccine.2022.05.026

Baniak, L. M., Luyster, F. S., Raible, C. A., McCray, E. E., & Strollo, P. J. (2021). COVID-19 Vaccine Hesitancy and Uptake among Nursing Staff during an Active Vaccine Rollout. *Vaccines*, *9*(8), 858. https://doi.org/10.3390/vaccines9080858

Dahie, H. A., Mohamoud, J. H., Adam, M. H., Garba, B., Dirie, N. I., Sh. Nur, M. A., & Mohamed, F. Y. (2022). COVID-19 Vaccine Coverage and Potential Drivers of Vaccine Uptake among Healthcare Workers in SOMALIA: A Cross-Sectional Study. *Vaccines*, *10*(7), 1116. https://doi.org/10.3390/vaccines10071116

Doran, J., Seyidov, N., Mehdiyev, S., Gon, G., Kissling, E., Herdman, T., Suleymanova, J., Rehse, A. P. C., Pebody, R., Katz, M. A., & Hagverdiyev, G. (2022). Factors associated with early uptake of COVID-19 vaccination among healthcare workers in Azerbaijan, 2021. *Influenza and Other Respiratory Viruses*, *16*(4), 626–631. https://doi.org/10.1111/irv.12978

Dubov, A., Distelberg, B. J., Abdul-Mutakabbir, J. C., Peteet, B., Roberts, L., Montgomery, S. B., Rockwood, N., Patel, P., Shoptaw, S., & Chrissian, A. A. (2022). Racial/Ethnic Variances in COVID-19 Inoculation among Southern California Healthcare Workers. *Vaccines*, *10*(8), 1331. https://doi.org/10.3390/vaccines10081331

Farah, W., Breeher, L., Shah, V., Hainy, C., Tommaso, C. P., & Swift, M. D. (2022). Disparities in COVID-19 vaccine uptake among health care workers. *Vaccine*, *40*(19), 2749–2754. https://doi.org/10.1016/j.vaccine.2022.03.045

Galanis, P., Moisoglou, I., Vraka, I., Siskou, O., Konstantakopoulou, O., Katsiroumpa, A., & Kaitelidou, D. (2022). Predictors of COVID-19 Vaccine Uptake in Healthcare Workers: A Cross-Sectional Study in Greece. *Journal of Occupational & Environmental Medicine*, *64*(4), e191–e196. https://doi.org/10.1097/JOM.0000000000002463

Gopaul, C. D., Ventour, D., & Thomas, D. (2022). *COVID-19 Vaccine Acceptance and Uptake Among Healthcare Workers in Trinidad & Tobago* [Preprint]. Public and Global Health. https://doi.org/10.1101/2022.05.09.22274854

Lucaccioni, H., Chakhunashvili, G., McKnight, C. J., Zardiashvili, T., Jorgensen, P., Pebody, R., Kissling, E., Katz, M. A., & Sanodze, L. (2022). Sociodemographic and Occupational Factors Associated with Low Early Uptake of COVID-19 Vaccine in Hospital-Based Healthcare Workers, Georgia, March–July 2021. *Vaccines*, *10*(8), 1197. https://doi.org/10.3390/vaccines10081197

Martin, C. A., Marshall, C., Patel, P., Goss, C., Jenkins, D. R., Ellwood, C., Barton, L., Price, A., Brunskill, N. J., Khunti, K., & Pareek, M. (2021). SARS-CoV-2 vaccine uptake in a multi-ethnic UK healthcare workforce: A cross-sectional study. *PLOS Medicine*, *18*(11), e1003823. https://doi.org/10.1371/journal.pmed.1003823

Oliver, K., Raut, A., Pierre, S., Silvera, L., Boulos, A., Gale, A., Baum, A., Chory, A., Davis, N. J., D'Souza, D., Freeman, A., Goytia, C., Hamilton, A., Horowitz, C., Islam, N., Jeavons, J., Knudsen, J., Li, S., Lupi, J., … Maru, D. (2022). Factors associated with COVID-19 vaccine receipt at two integrated healthcare systems in New York City: A cross-sectional study of healthcare workers. *BMJ Open*, *12*(1), e053641. https://doi.org/10.1136/bmjopen-2021-053641

Rikitu Terefa, D., Shama, A. T., Feyisa, B. R., Ewunetu Desisa, A., Geta, E. T., Chego Cheme, M., & Tamiru Edosa, A. (2021). COVID-19 Vaccine Uptake and Associated Factors Among Health Professionals in Ethiopia. *Infection and Drug Resistance*, *14*, 5531–5541. https://doi.org/10.2147/IDR.S344647

Xu, B., Gao, X., Zhang, X., Hu, Y., Yang, H., & Zhou, Y.-H. (2021). Real-World Acceptance of COVID-19 Vaccines among Healthcare Workers in Perinatal Medicine in China. *Vaccines*, *9*(7), 704. https://doi.org/10.3390/vaccines9070704

Zdravkovic, M., Popadic, V., Nikolic, V., Klasnja, S., Brajkovic, M., Manojlovic, A., Nikolic, N., & Markovic-Denic, L. (2022). COVID-19 Vaccination Willingness and Vaccine Uptake among Healthcare Workers: A Single-Center Experience. *Vaccines*, *10*(4), 500. https://doi.org/10.3390/vaccines10040500
