# Supplementary figures and images for: COVID-19 Vaccine Uptake among Healthcare Workers: A Systematic Review and Meta-Analysis
Source: Vaccines (Basel). 2022 Sep 29;10(10):1637. doi: 10.3390/vaccines10101637 (PMC9610263; doi:10.3390/vaccines10101637)

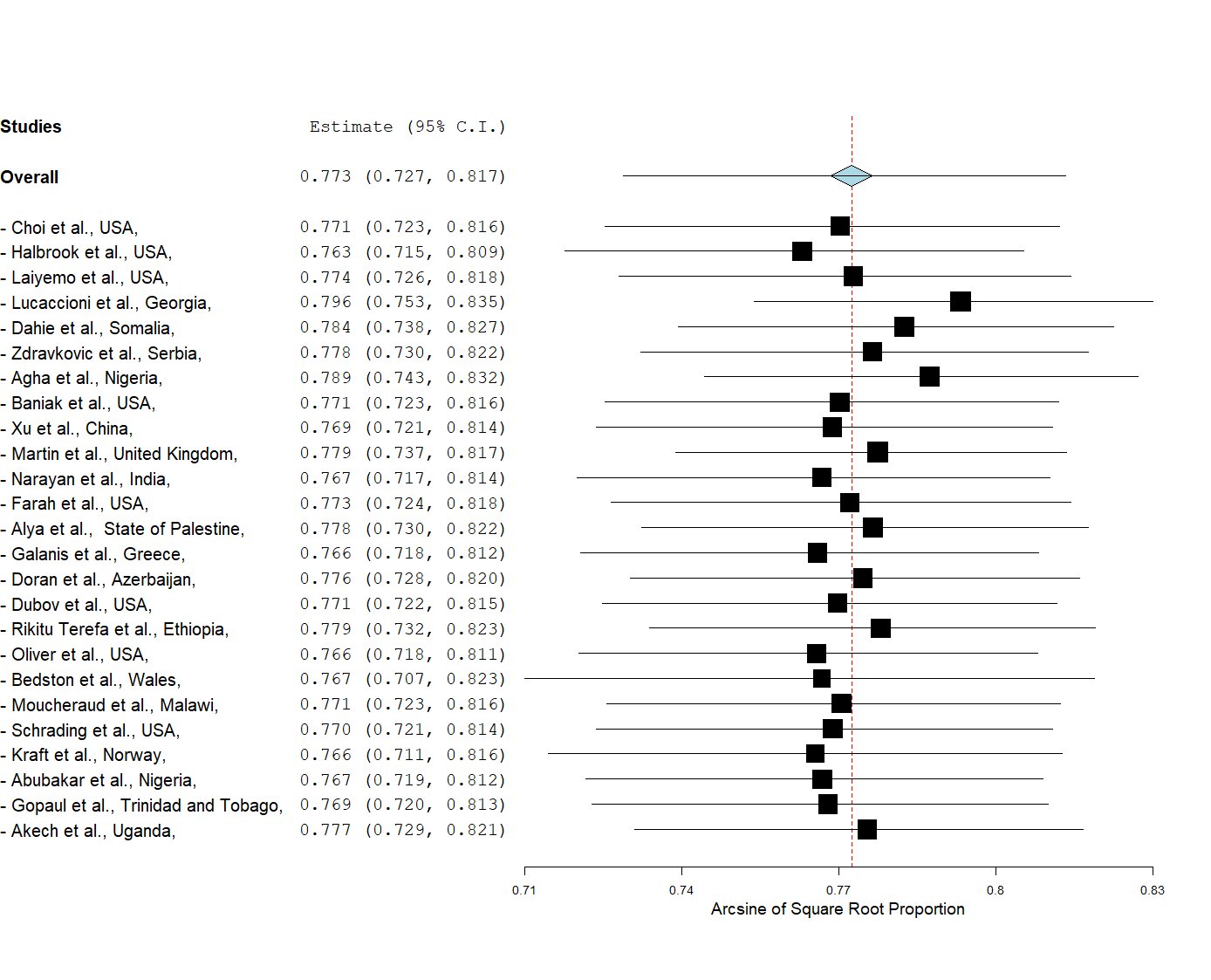

Supplement: Supplementary file 1 [file vaccines-10-01637-s001.zip › Supplementary Figure S1.jpg]

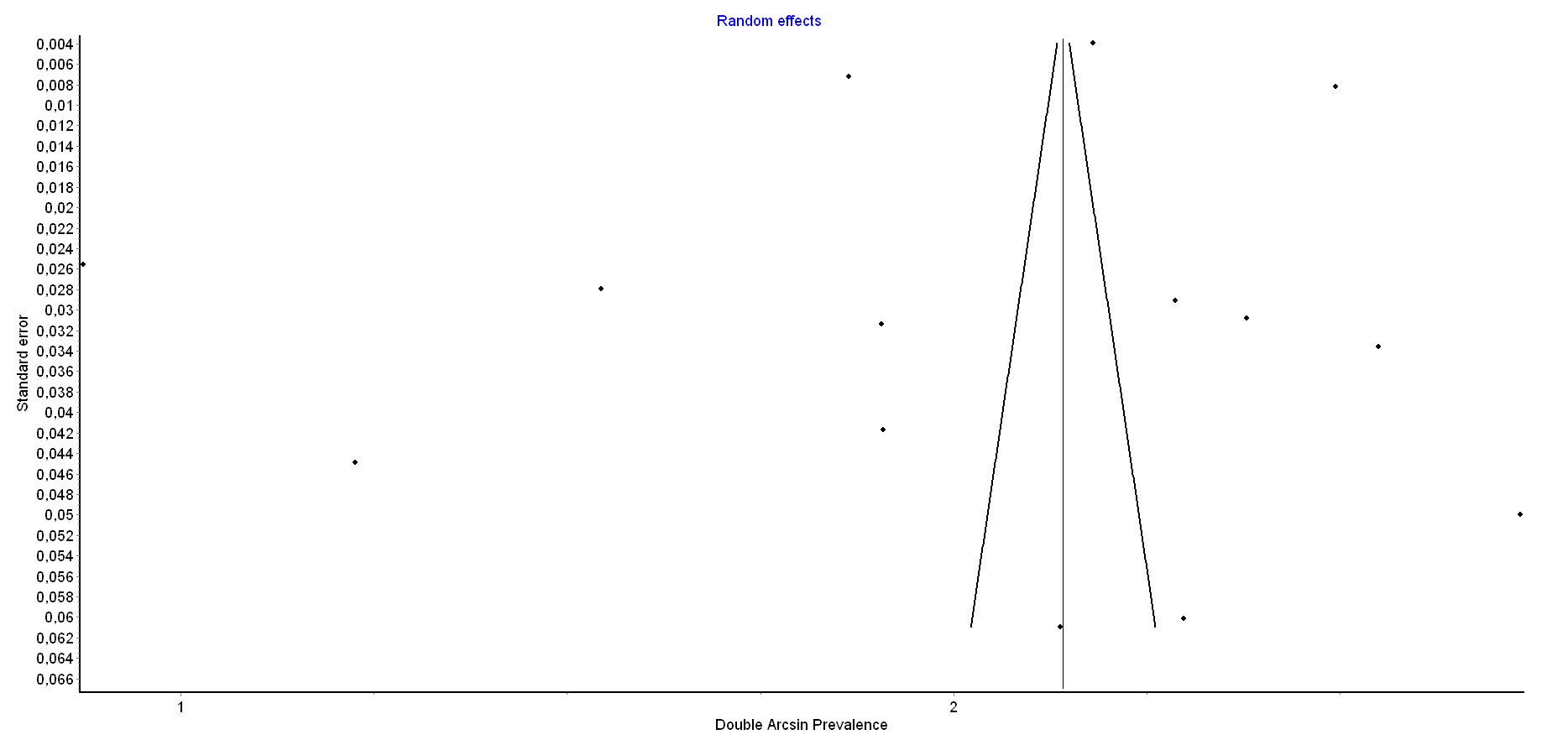

Supplement: Supplementary file 1 [file vaccines-10-01637-s001.zip › Supplementary Figure S2.jpg]

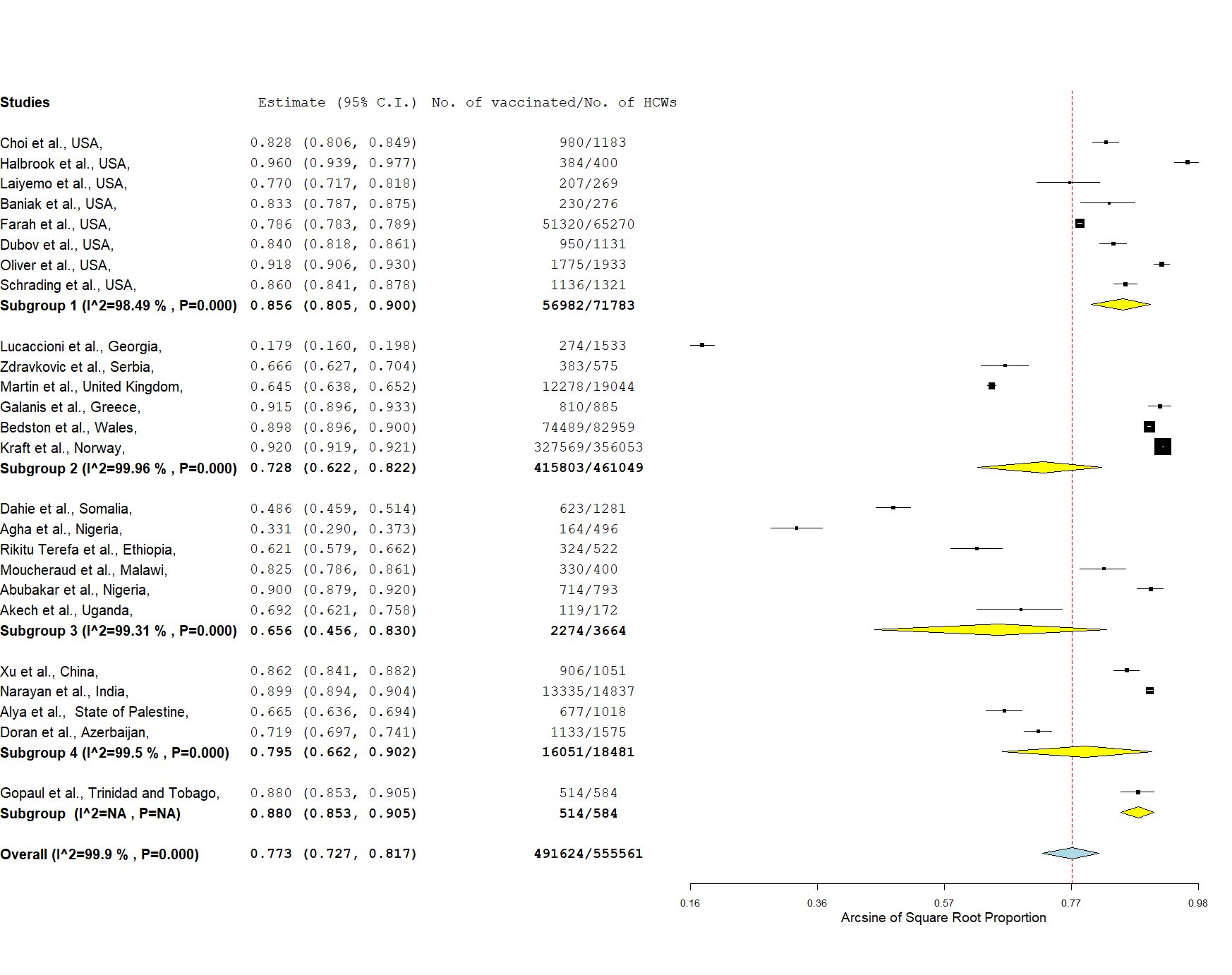

Supplement: Supplementary file 1 [file vaccines-10-01637-s001.zip › Supplementary Figure S3.jpg]

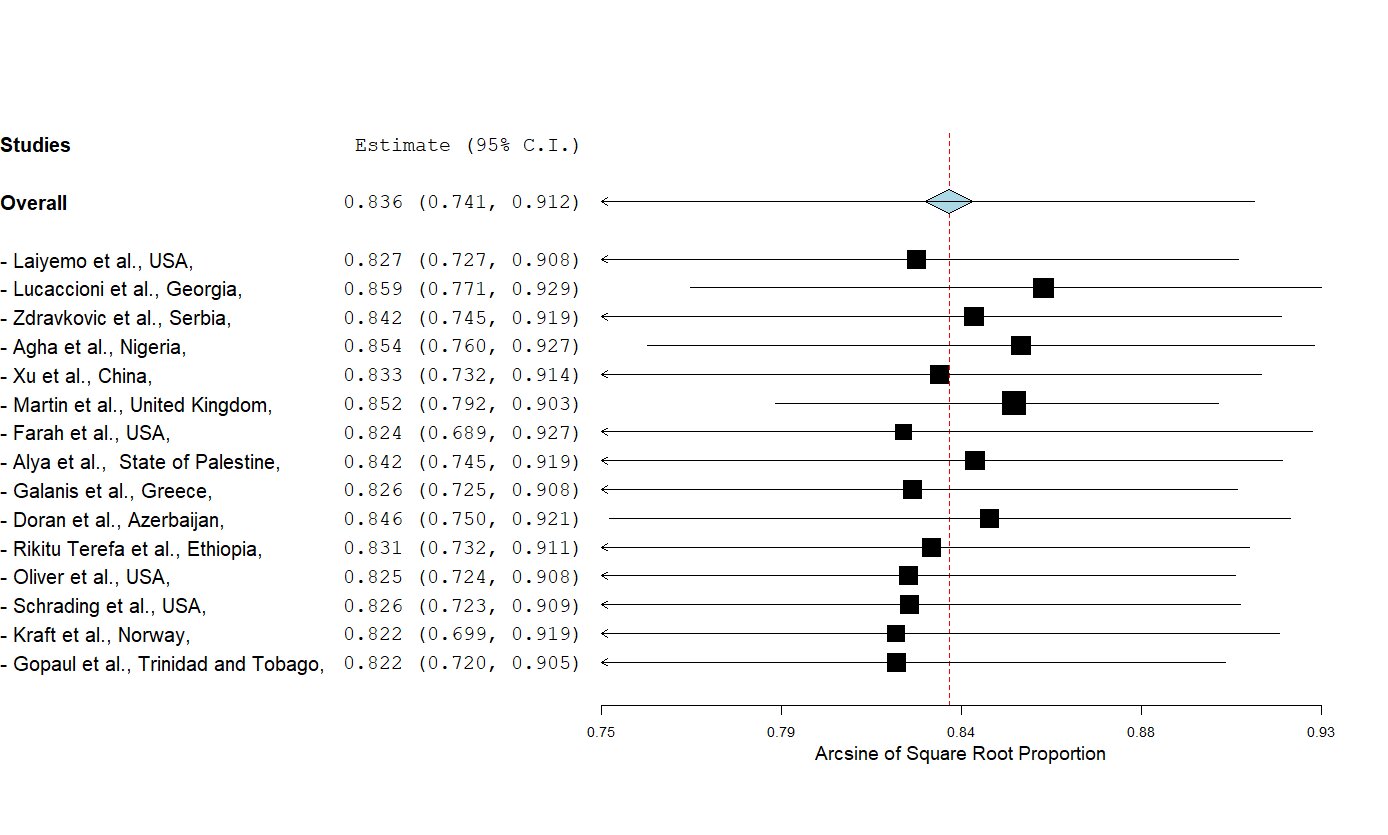

Supplement: Supplementary file 1 [file vaccines-10-01637-s001.zip › Supplementary Figure S4.jpg]

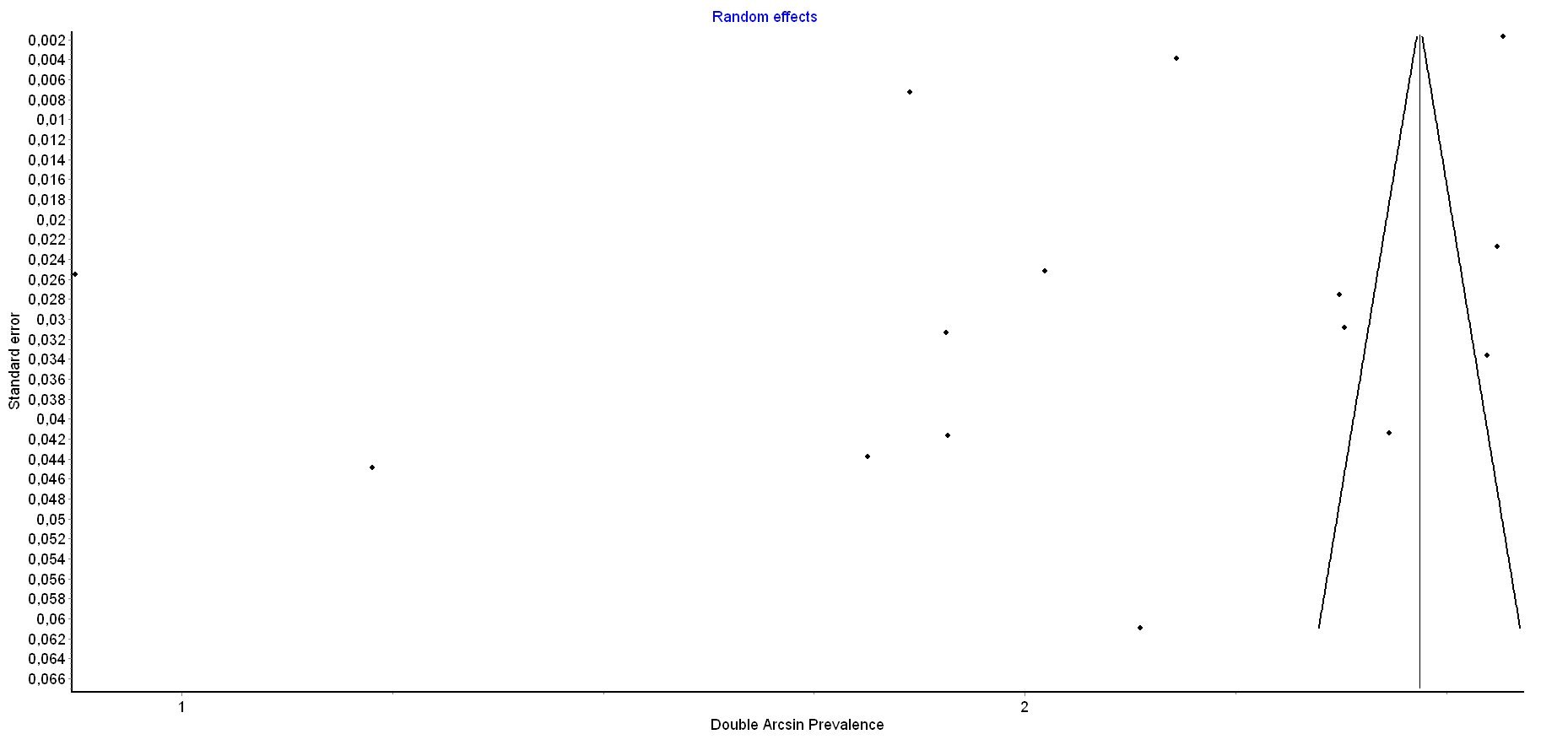

Supplement: Supplementary file 1 [file vaccines-10-01637-s001.zip › Supplementary Figure S5.jpg]

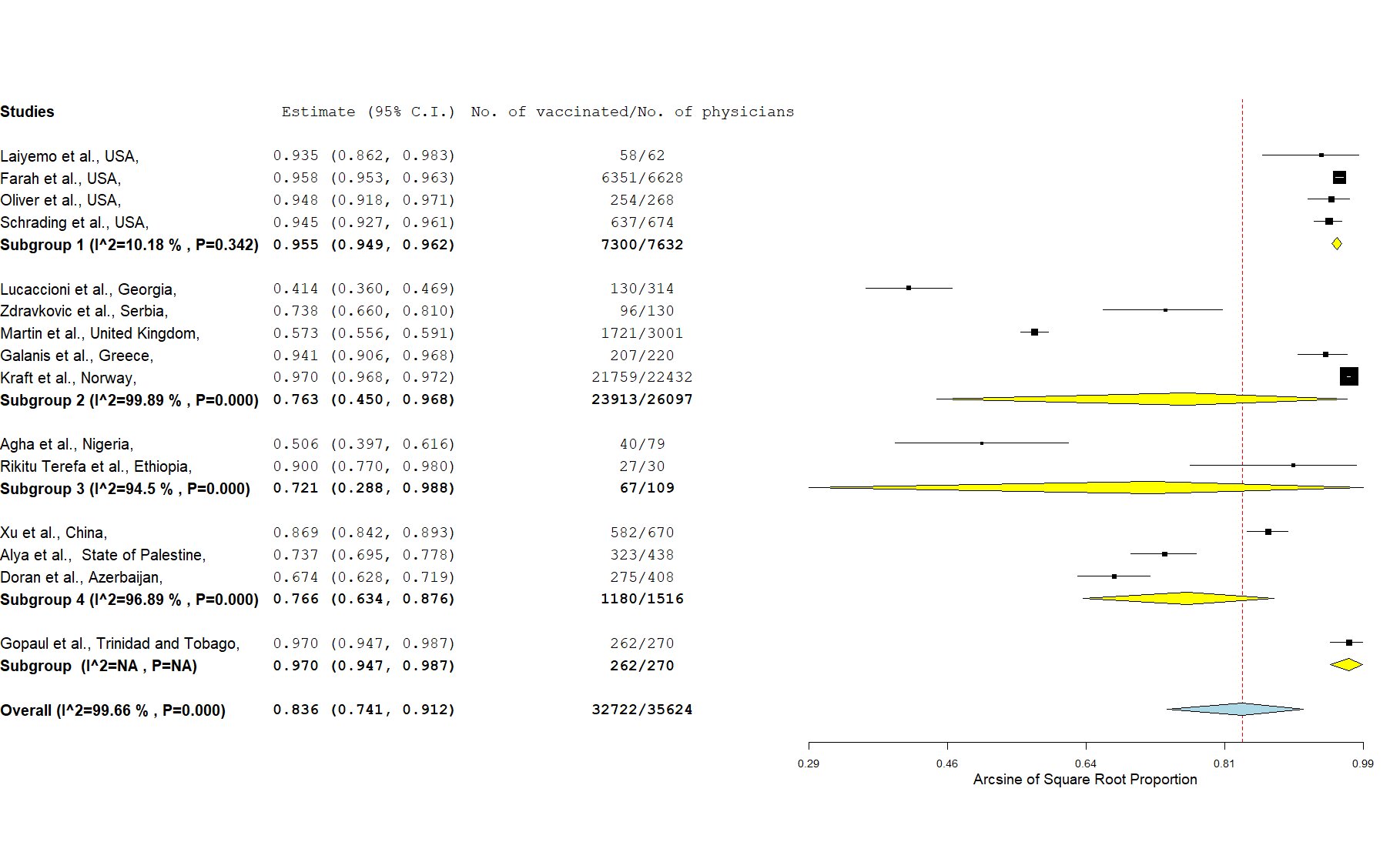

Supplement: Supplementary file 1 [file vaccines-10-01637-s001.zip › Supplementary Figure S6.jpg]

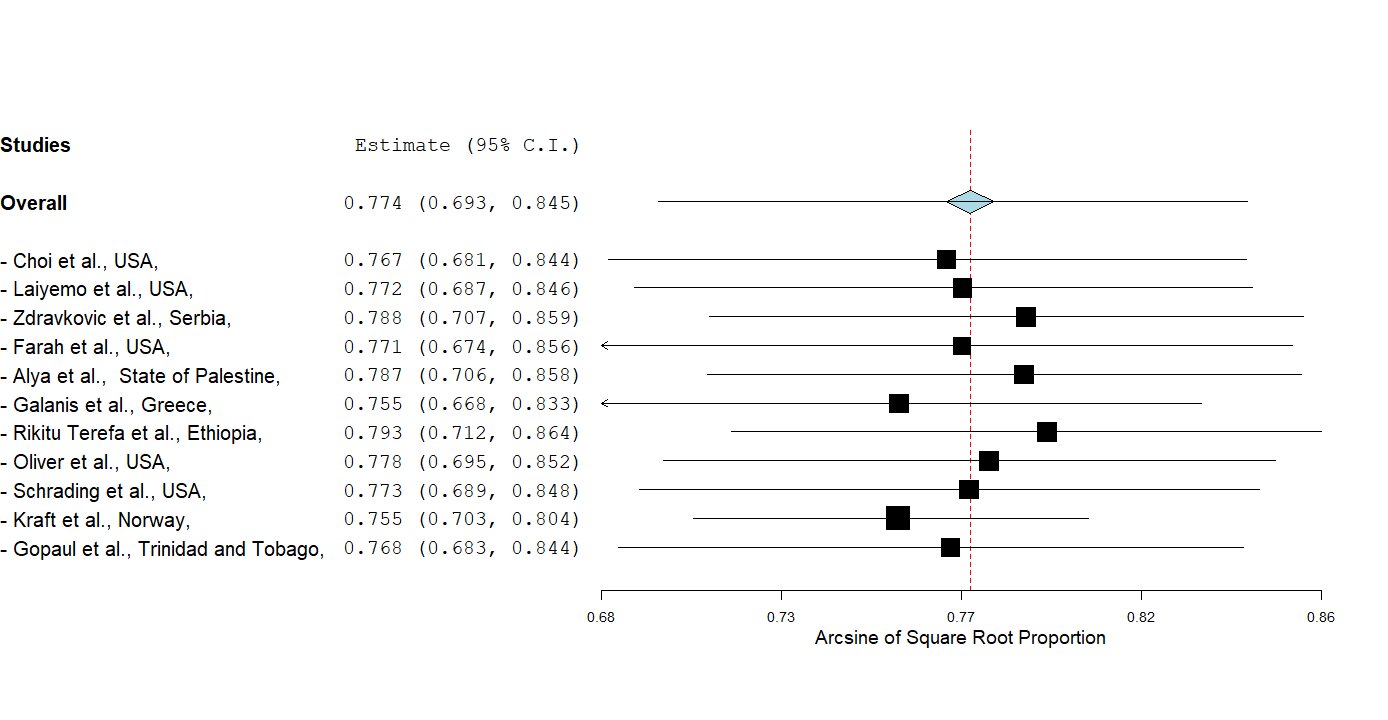

Supplement: Supplementary file 1 [file vaccines-10-01637-s001.zip › Supplementary Figure S7.jpg]

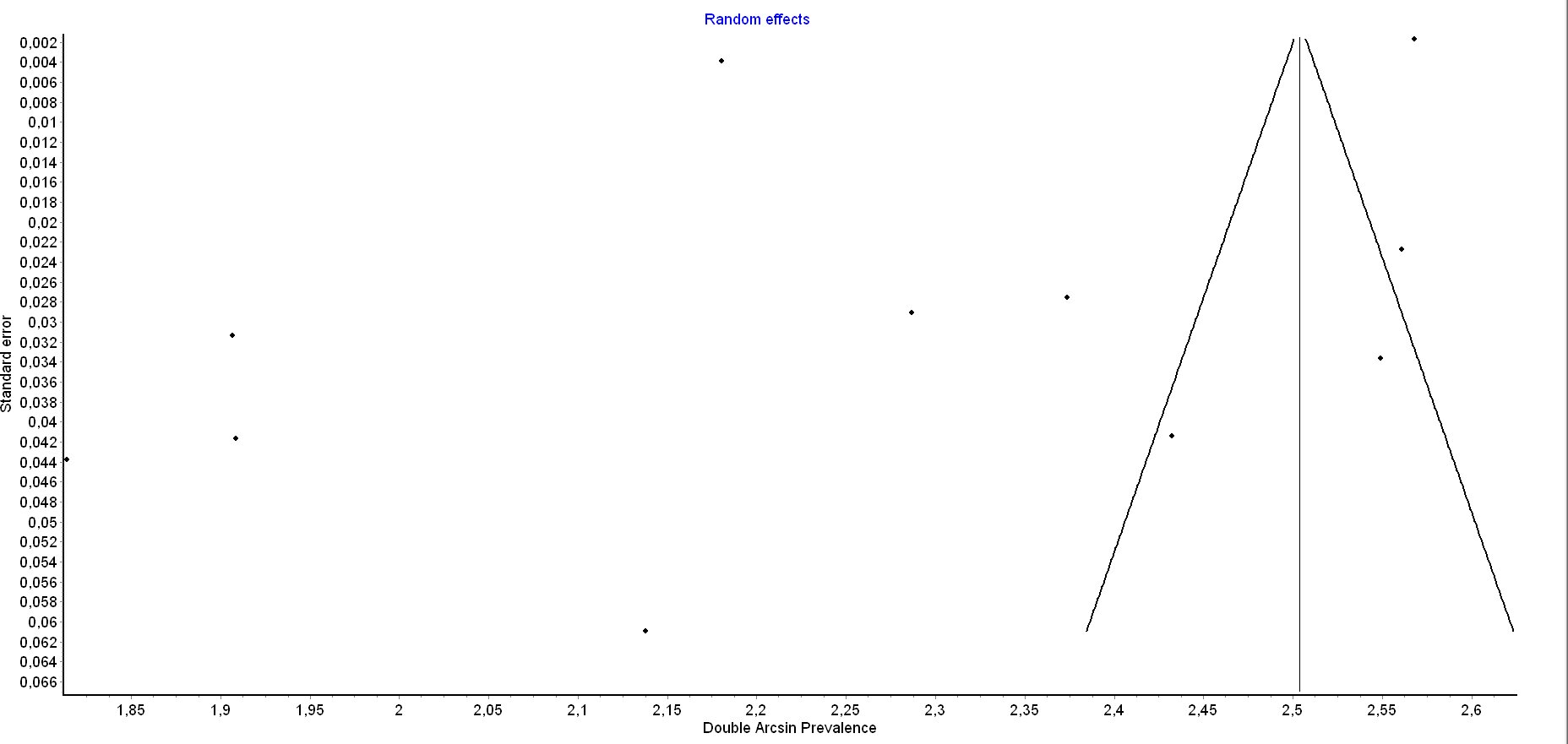

Supplement: Supplementary file 1 [file vaccines-10-01637-s001.zip › Supplementary Figure S8.jpg]

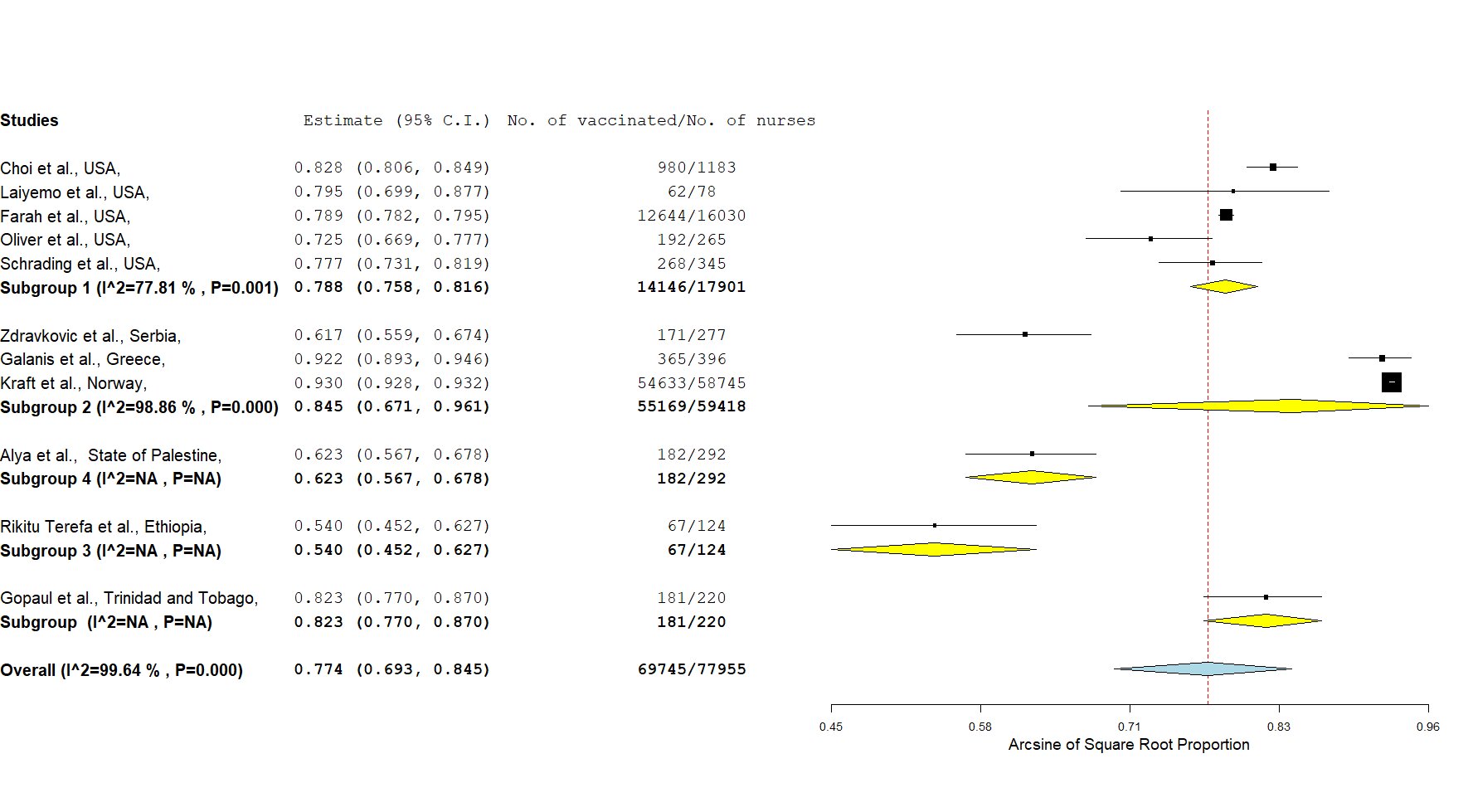

Supplement: Supplementary file 1 [file vaccines-10-01637-s001.zip › Supplementary Figure S9.jpg]
